# Mechanical memory of confinement pressure governs expansion size in epithelial monolayers

**DOI:** 10.26508/lsa.202603831

**Published:** 2026-08-20

**Authors:** Linn Engström, Simon K Schnyder, Johannes K Ahnlide, Valeriia Grudtsyna, Martijn Gloerich, Pontus Nordenfelt, Amin Doostmohammadi, Vinay S Swaminathan

**Affiliations:** 1 Division of Oncology, Department of Clinical Sciences, Lund University , Lund, Sweden; 2 Wallenberg Centre for Molecular Medicine, Lund University , Lund, Sweden; 3 Institute of Industrial Science, The University of Tokyo, Tokyo, Japan; 4 Theoretical Sciences Visiting Program, Okinawa Institute of Science and Technology Graduate University , Onna, Japan; 5 Division of Infection Medicine, Department of Clinical Sciences, Lund University , Lund, Sweden; 6 Centre for Molecular Medicine, UMC Utrecht, Utrecht, Netherlands; 7 Niels Bohr Institute, University of Copenhagen , Copenhagen, Denmark

## Abstract

Epithelial tissues undergo rapid expansion during development, repair, and morphogenesis, yet how tissue-scale growth is coordinated to re-establish homeostasis remains unclear. Here, we show that large epithelial monolayers confined at a wide range of initial densities and mechanochemical states robustly converge to the same final size and density upon release, despite differences in initial cell size, YAP activity, and cell number dynamics. To investigate the underlying mechanism, we combined quantitative experiments with a mechanochemical agent–based model in which mechanical pressure arising from confinement acts as a tissue-scale signal that modulates intracellular cell-cycle activity over time. Using this framework, we show that transient mechanical relaxation during confinement selectively elevates cell-cycle activity in higher density tissues at the time of release, accelerating early expansion without disrupting final homeostatic outcomes. Together, these results reveal how epithelial tissues coordinate collective growth and robustly restore homeostasis during expansion.

## Abstract (precis)

Confined epithelial tissues encode pressure history into cell-cycle activity, enabling colonies of different densities to converge on the same size and density upon release.

## Introduction

Maintenance of epithelial homeostasis during tissue expansion in embryonic development and wound healing is essential for normal tissue function and repair. This requires tissues to rapidly adapt to increases in size by spatiotemporally regulating proliferation while maintaining appropriate cell numbers and density (Friedl & Gilmour, 2009; Blanpain & Fuchs, 2014; Macara et al, 2014; Tai et al, 2019; Franco et al, 2019). Although cell-scale functions such as division and extrusion in this context are well described, how large epithelial tissues composed of asynchronously cycling cells and diverse morphologies coordinate these behaviours across length scales to continuously maintain homeostasis during expansion remains poorly understood (Eisenhoffer et al, 2012; Puliafito et al, 2012; Chiba et al, 2016; Kocgozlu et al, 2016; Mayor & Etienne-Manneville, 2016; Saw et al, 2017; Franco et al, 2019; Heinrich et al, 2020).

Mechanical forces act as regulators of proliferation in epithelial tissues by activating several downstream mechanotransduction pathways (Dupont et al, 2011; Aragona et al, 2013; Uroz et al, 2018; Di Meglio et al, 2022; Höllring et al, 2024). Stretching can activate YAP, TAZ, and β-catenin to promote cell-cycle entry and progression, whereas mechanosensitive channels such as Piezo1 respond to both tensile and compressive forces to modulate cell behaviour (Huang & Ingber, 1999; Coste et al, 2010; Dupont et al, 2011; Aragona et al, 2013; Benham-Pyle et al, 2015; Gudipaty et al, 2017; Saw et al, 2017; Tai et al, 2019; Di Meglio et al, 2022). In particular, mechanical activation of YAP has been shown to regulate cell proliferation during lung branching, and renewal of the intestinal epithelium and epidermal tissues establishing YAP as a central mediator of local proliferative responses to changes in mechanical stress associated with local cell density (McClatchey & Yap, 2012; Imajo et al, 2014; Streichan et al, 2014; Lin et al, 2017). However, both mechanical stress and cell density vary spatially across expanding tissues, and it remains unclear how such heterogeneous local cues are integrated to activate signals such as YAP/TAZ and coordinate proliferation across the tissue.

Previous experimental and computational studies have shown that coupling proliferation to a tissue pressure, emerging from the interplay between proliferation and tissue expansion, can account for the maintenance of density homeostasis (Basan et al, 2009, 2011; Marel et al, 2014; Höllring et al, 2023). However, these models typically idealize intracellular regulation and rely on simplified forms of mechanosensitivity, such as instantaneous reductions in proliferation rate as a function of density, force, or pressure. Thus, it still remains unclear whether and how cells integrate mechanical history to regulate proliferation, how this regulation responds to perturbations, and which intracellular mechanisms confer robustness to such feedback.

To address these questions, we combined controlled tissue-scale experiments with computational modelling. We grew large two-dimensional epithelial monolayers at defined initial densities under circular confinement using polydimethylsiloxane (PDMS) stencils and quantified both cell-scale and tissue-scale properties during subsequent expansion using high-resolution imaging and analysis pipelines. This enabled systematic comparison of expansion dynamics across a wide range of starting densities and mechanochemical states. Using this experimental framework, we find that epithelial tissues robustly re-establish density homeostasis during expansion despite large differences in initial density, cell size, and YAP activity at the time of confinement release. Across conditions, colonies expand to similar final sizes and densities, indicating the presence of tissue-scale coordination mechanisms that buffer local heterogeneity. To investigate the basis of this coordination, we employed an agent-based model incorporating mechanochemical feedback between mechanical pressure and intracellular regulatory state, providing a framework to explore how mechanical history may influence collective tissue behaviour during expansion.

## Results

### Confined epithelial colonies exhibit two density-dependent YAP regimes before expansion

**Figure 1.**
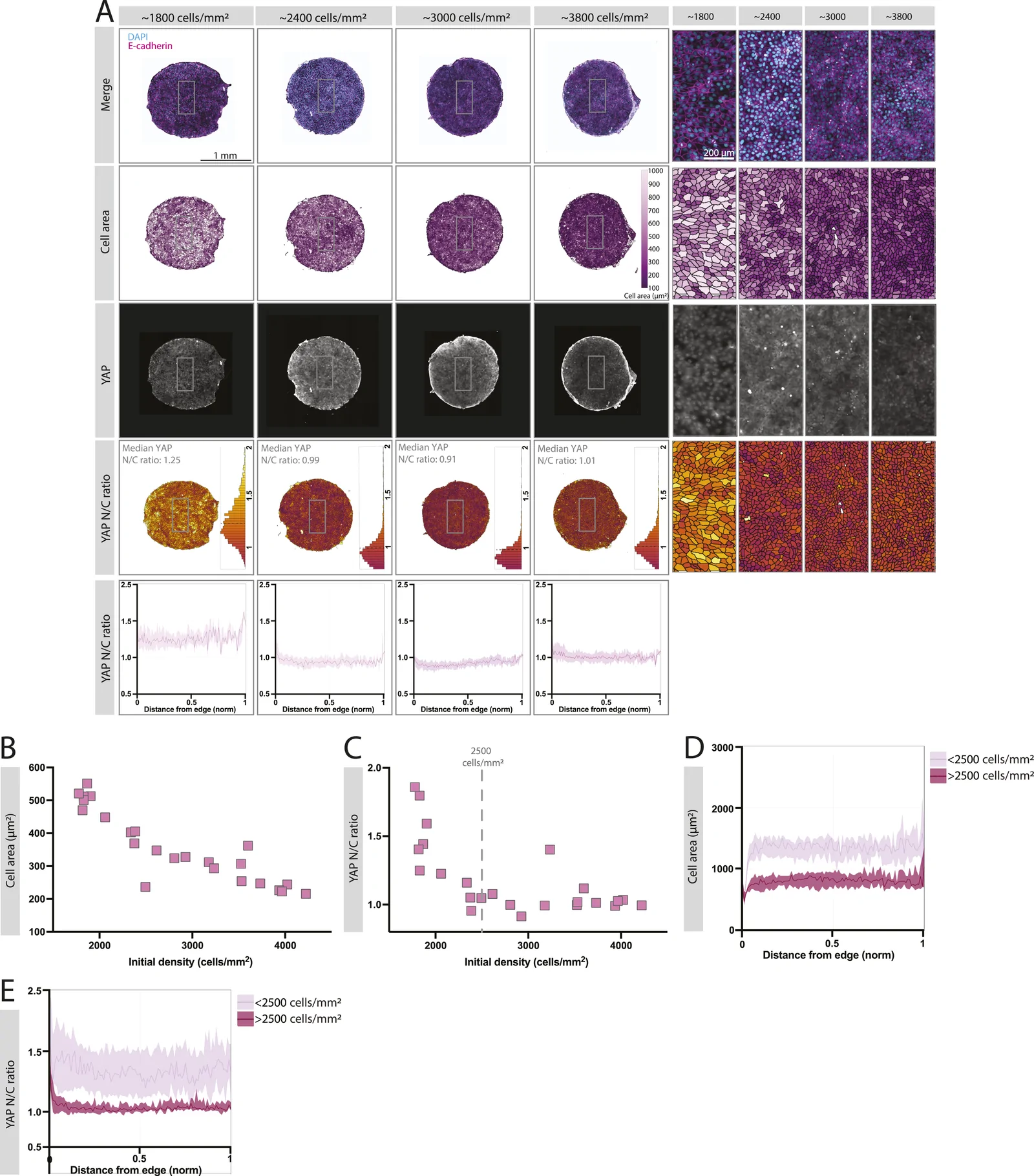
Confined epithelial colonies exhibit two density-dependent YAP regimes prior to expansion. **(A)** Representative images of DAPI/E-cadherin merge, cell area colour maps, YAP channel, and YAP N/C colour maps of colonies of varying densities ranging from ~1800 to ~3800 cells/mm^2^. Images taken immediately after removal of stencil, color-coded for cell area and YAP N/C ratio. Representative histograms display the population spread for YAP N/C ratios and colour scale from 0.8 to 2. The dashed lines indicate the median YAP N/C ratio for the colony. Radial spatial plots displaying median YAP N/C ratio of example colonies. Thicker line represents median YAP across colony, with shaded error bars representing interquartile range (IQR) of these. X-axis represents the distance from edge to centre, which has been normalized to arbitrary values. Scale bar (whole colony images), 1 mm. Scale bar (zoom in images) 200 µm. **(B)** Cell area in µm² as a factor of initial density at 0 h across the density range ~1700 cells/mm^2 ^to ~4500 cells/mm^2^ (*n* = 24 colonies from at least eight individual experiments). **(C)** YAP N/C ratio as a factor of initial density at 0 h across the density range ~1700 cells/mm^2 ^to ~4500 cells/mm^2^ (*n* = 24 colonies from at least eight individual experiments). Dashed line indicates cut off from the YAP-dependent density range (~1700–2500 cells/mm^2^) to the YAP-independent density range (~2500–4500 cells/mm^2^). **(D)** Radial spatial plots displaying median cell area of colonies, divided into colonies with a density <2500 cells/mm^2^ and >2500 cells/mm^2^. Thicker line represents median cell area across colony, with shaded error bars representing interquartile range (IQR) of these. X-axis represents the distance from edge to centre, which has been normalized to arbitrary values. **(E)** Radial spatial plots displaying median YAP N/C ratio of colonies, divided into colonies with a density <2500 cells/mm^2^ and >2500 cells/mm^2^. Thicker line represents median YAP ratio across colony, with shaded error bars representing interquartile range (IQR) of these. X-axis represents the distance from edge to centre, which has been normalized to arbitrary values.

**Figure S1.**
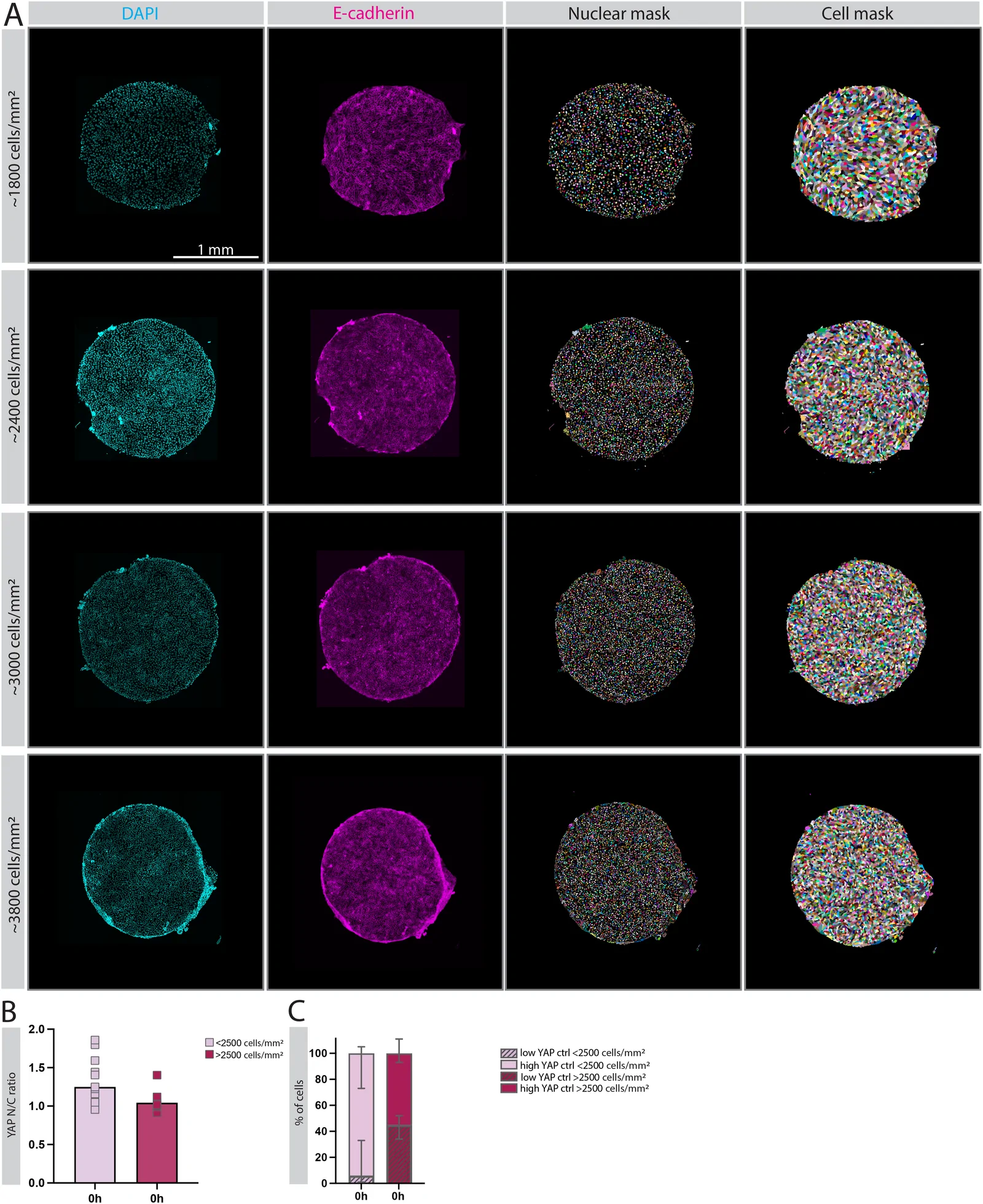
Confined epithelial colonies exhibit two density-dependent YAP regimes prior to expansion. **(A)** Stitched confocal images of DAPI (cyan) and E-cadherin (magenta), YAP (grey) channels, as well as cell and nuclei segmentation masks in representative low-, intermediate- and-high density colonies with a diameter of 1.5 mm at initiation of experiments (0 h). Scale bar, 0.5 mm. **(B)** YAP N/C ratio of colonies <2500 and >2500 cells/mm^2 ^imaged and measured after removal of stencil (*n* = 11 <2500 cells/mm^2^ and 13 >2500 cells/mm^2^ colonies from at least eight individual experiments). **(C)** Percentage of cells with high or low YAP N/C ratio of colonies <2500 and >2500 cells/mm^2 ^imaged and measured after removal of stencil (*n* = 11 <2500 cells/mm^2^ and 13 >2500 cells/mm^2^ colonies from at least eight individual experiments).

To understand the physical mechanisms that regulate the re-establishment of homeostasis after tissue expansion, we first sought to map the relationship between tissue density and the mechanochemical state of confined epithelial monolayers at the onset of expansion. Because YAP activity has previously been shown to be a key indicator and regulator of proliferative potential and mechanotransduction in epithelial tissues, we used the nuclear-to-cytoplasmic ratio of YAP (YAP N/C) as a quantitative readout of tissue mechanochemical state (Dupont et al, 2011; Aragona et al, 2013; Benham-Pyle et al, 2015; Chiba et al, 2016; Saw et al, 2017). We seeded E-cadherin-RFP–expressing Madin-Darby Canine Kidney (MDCK) cells within 1.5-mm-diameter circular PDMS stencils on fibronectin-coated glass coverslips (Figs 1A and S1A).

Cells were seeded at varying numbers, which led to confined monolayers with densities ranging from ∼1,700 to ∼4,200 cells/mm^2^ within the stencils. Upon formation of a complete monolayer, stencils were removed, and the colonies were immediately fixed, stained for YAP, F-actin, and DAPI, and imaged using high-resolution confocal microscopy. We developed in-house analysis pipelines to generate stitched, single-cell resolved maps of the entire colony, enabling precise quantification of cell number, cell area, and YAP N/C for all cells within the colony (Figs 1A and S1A).

As expected, cell area heat maps showed clear differences across the range of initial colony densities, with a marked reduction in overall cell size in high-density colonies compared with lower ones (Fig 1A). Quantification of median cell area for each colony confirmed a monotonically decreasing trend across the density range (Fig 1B). In contrast, heat maps of YAP N/C revealed a non-linear dependence on colony density (Fig 1A and B). Quantification of median YAP N/C as a function of colony density identified two distinct regimes; in colonies with densities <∼2,500 cells/mm^2^, median YAP N/C started high and rapidly decreased with increasing density, whereas in colonies >∼2,500 cells/mm^2^, YAP N/C remained low and was insensitive to further increases in colony density (Figs 1C and S1B and C).

We next asked whether this density-dependent behaviour of cell area and YAP N/C reflected uniform regulation across cells within a colony or instead arose from spatial or cell-to-cell heterogeneity within each density regime. To address this, we analysed YAP N/C distributions and performed spatial analysis of both cell area and YAP N/C within individual colonies. Histograms of YAP N/C in lower density colonies <∼2,500 cells/mm^2^ were broad, with a substantial fraction of cells exhibiting high YAP N/C values, whereas distributions in higher density colonies >2,500 cells/mm^2^ were significantly narrower and centred around low median YAP N/C values. Spatial analysis further revealed that in low-density colonies, YAP N/C and cell area were uniformly elevated across the colony with no gradient towards the centre, whereas high-density colonies exhibited a localized increase in YAP N/C restricted to the immediate colony edge, with uniformly low YAP N/C and reduced cell area throughout the colony interior (Fig 1D and E).

Together, these results show that YAP activity in confined epithelial monolayers varies non-linearly with cell density before expansion, defining distinct density ranges with different sensitivities to crowding. YAP activity is uniformly elevated at lower densities and decreases with increasing overall colony density, where at higher densities, it is globally suppressed and only weakly sensitive to further crowding.

### Tissue-wide cell number regulation couples epithelial expansion size to re-establishment of homeostasis

**Figure 2.**
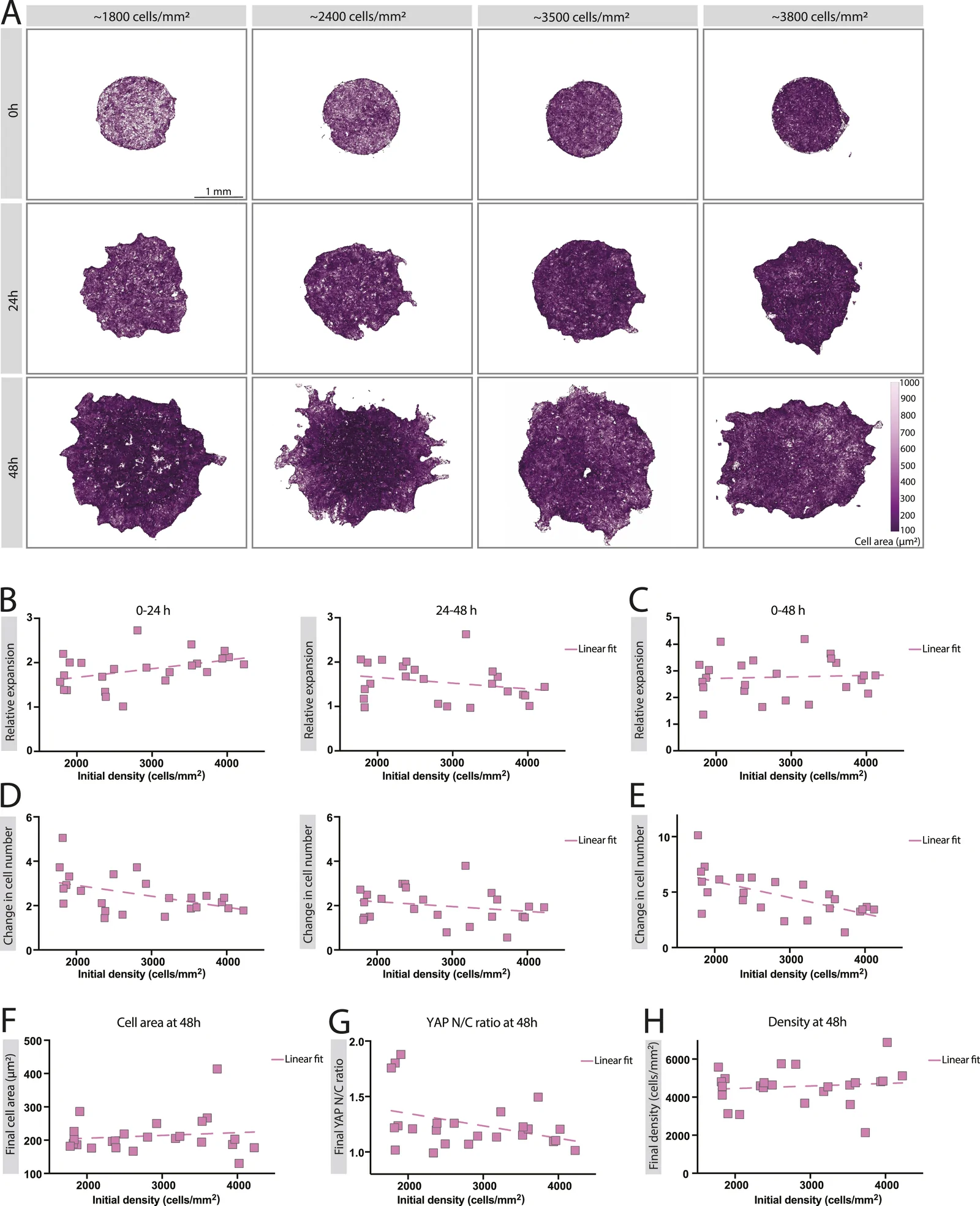
Tissue-wide cell number regulation couples epithelial expansion size to re-establishment of homeostasis. **(A)** Cell area colour maps of representative epithelial colonies with an initial diameter of 1.5 mm of varying densities ranging from ~1800 to ~3800 cells/mm^2^. Images acquired after 0, 24 and 48 h of expansion, segmented, and colour-coded according to cell area and with cell membrane outlines drawn as overlay. Scale bar, 1 mm. **(B)** Relative expansion from 0 to 24, and 24 to 48 h as a factor of initial density at 0 h across the density range ~1700 cells/mm^2 ^to ~4500 cells/mm^2^ (*n *= 24 colonies from at least eight individual experiments). Relative expansion is defined as the fold change in colony area from start of expansion. Dashed line represents linear fit. **(C)** Relative expansion from 0 to 48 h as a factor of initial density at 0 h across the density range ~1700 cells/mm^2 ^to ~4500 cells/mm^2^ (*n *= 24 colonies from at least eight individual experiments). Relative expansion is defined as the fold change in colony area from start of expansion. Dashed line represents linear fit. **(D)** Change in cell number from 0 to 24, and 24 to 48 h as a factor of initial density at 0 h across the density range ~1700 cells/mm^2 ^to ~4500 cells/mm^2^ (*n *= 24 colonies from at least eight individual experiments). Dashed line represents linear fit. **(E)** Change in cell number from 0 to 48 h as a factor of initial density at 0 h across the density range ~1700 cells/mm^2 ^to ~4500 cells/mm^2^ (*n *= 24 colonies from at least eight individual experiments). Dashed line represents linear fit. **(F)** Final cell area at 48 h as a factor of the initial density at 0 h of colonies across the density range ~1700 cells/mm^2 ^to ~4500 cells/mm^2^ (*n *= 24 colonies from at least eight individual experiments). Dashed line represents linear fit. **(G)** Final YAP N/C ratio at 48 h as a factor of the initial density at 0 h of colonies across the density range ~1700 cells/mm^2 ^to ~4500 cells/mm^2^ (*n *= 24 colonies from at least eight individual experiments). Dashed line represents linear fit. **(H)** Final density at 48 h as a factor of the initial density at 0 h of colonies across the density range ~1700 cells/mm^2 ^to ~4500 cells/mm^2^ (*n *= 24 colonies from at least eight individual experiments). Dashed line represents linear fit.

**Figure S2.**
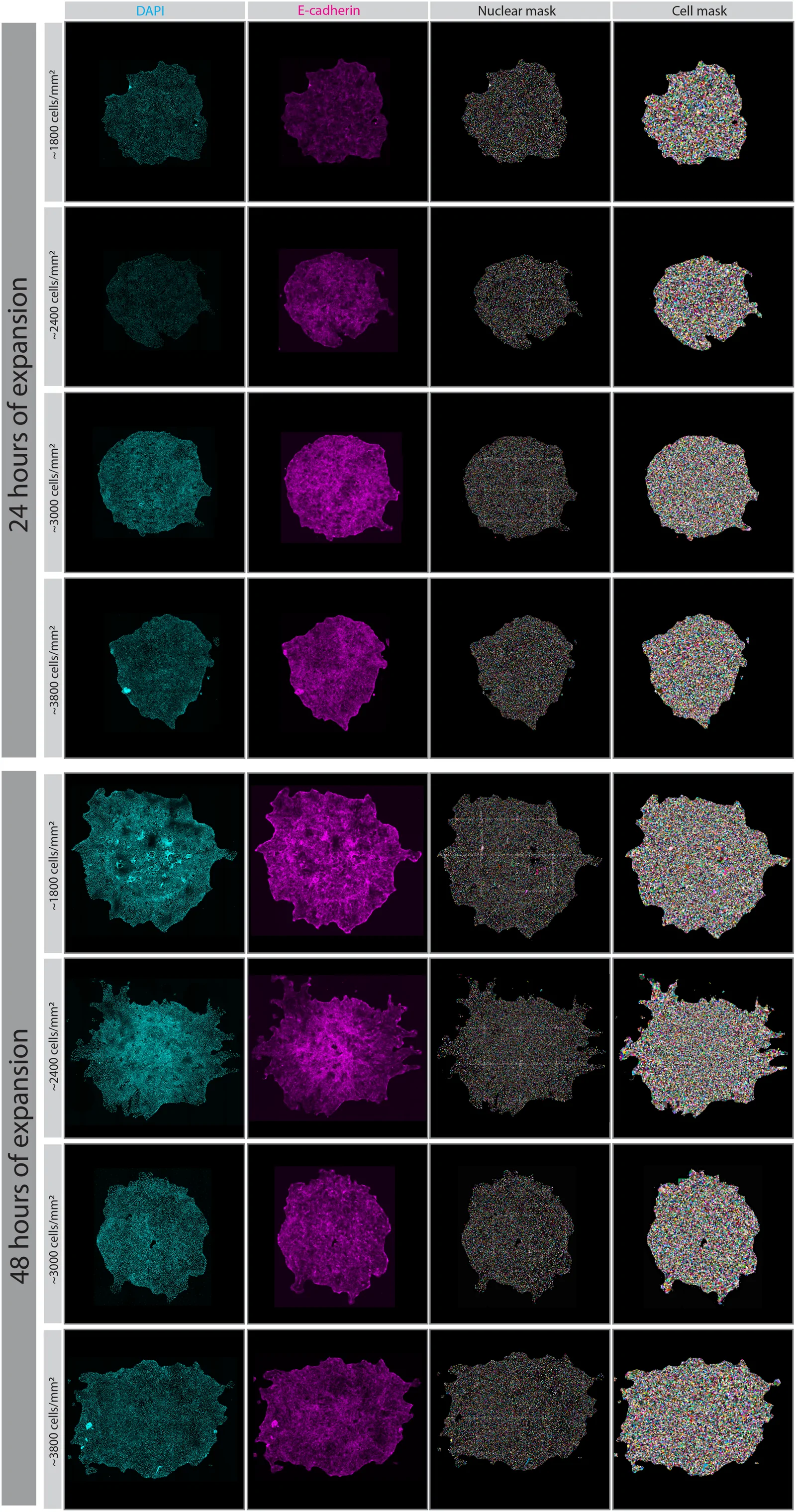
Tissue-wide cell number regulation couples epithelial expansion size to re-establishment of homeostasis. Stitched large confocal images of DAPI (cyan) and E-cadherin (magenta) channels and their respective segmented nuclear and cell masks of colonies in densities ranging from ~1800 to ~3800 cells/mm^2^. Images acquired after 24 and 48 h of expansion, segmented and colour-coded according to cell area and with cell and after 24 and 48 h of expansion.

Building on the non-linear density dependence of YAP activity and the emergence of distinct mechanochemical states before expansion, we next investigated how these states influence epithelial expansion after confinement release. To do so, we now allowed the monolayers confined across the density range to expand for 24 or 48 h before fixing, staining, and further analysis (Figs 2A and S2).

In addition to quantifying tissue expansion at 24 and 48 h, we measured changes in cell area, YAP N/C, and total cell number across the range of densities (Fig 2B–G). We found that for the first 24 h after confinement release, colonies originating from higher initial densities exhibited slightly greater tissue expansion, whereas colonies originating from lower initial densities showed a larger increase in the total cell number over the same period, consistent with their higher YAP N/C at the time of release (Fig 2B and D). During the subsequent 24 h, differences in expansion across densities were modest, whereas colonies originating from lower initial densities continued to show a greater net increase in cell number which accompanied slightly higher expansion (Fig 2B and D). As a result, despite differences in early expansion and cell number dynamics, colonies across the full initial density range reached comparable final extents of expansion after 48 h, resulting in overall tissue expansion to be independent of initial density (Fig 2C). In contrast, cumulative changes in cell number over the 48-h period remained density-dependent, with colonies starting at lower initial densities exhibiting larger net increases in cell number (Fig 2E). Together, these observations indicate that epithelial colonies converge to a similar expanded size after confinement release, whereas differences in cell number dynamics persist as a function of initial density, reflecting density-dependent regulation of cell number rather than differences in overall tissue expansion. These results show that YAP activity in confined epithelial monolayers varies non-linearly with cell density before expansion, defining distinct density ranges with different sensitivities to crowding. YAP activity is uniformly elevated at lower densities and decreases with increasing overall colony density, where at higher densities, it is globally suppressed and only weakly sensitive to further crowding.

Given these density-dependent differences in cell number dynamics coupled with overall similar expansion size, we next examined whether epithelial colonies converged to a common cell-scale mechanochemical state after 48 h of expansion. This analysis showed that despite significant starting density-dependent differences in cell number, cell area, and YAP activity at the onset of expansion post-confinement, all of these parameters converged to similar values after 48 h, leading to an overall convergence of all expanding colonies to a final density of ∼4,500 cells/mm^2^ (Fig 2F–H).

Together, these results show that epithelial expansion is dynamically coupled to regulation of mechanochemical state and cell number, enabling robust, density-independent expansion and convergence to a common homeostatic state.

### Agent-based modelling reveals mechanochemical feedback drives tissue-scale pressure regulation and density homeostasis in expanding epithelial colonies

**Figure 3.**
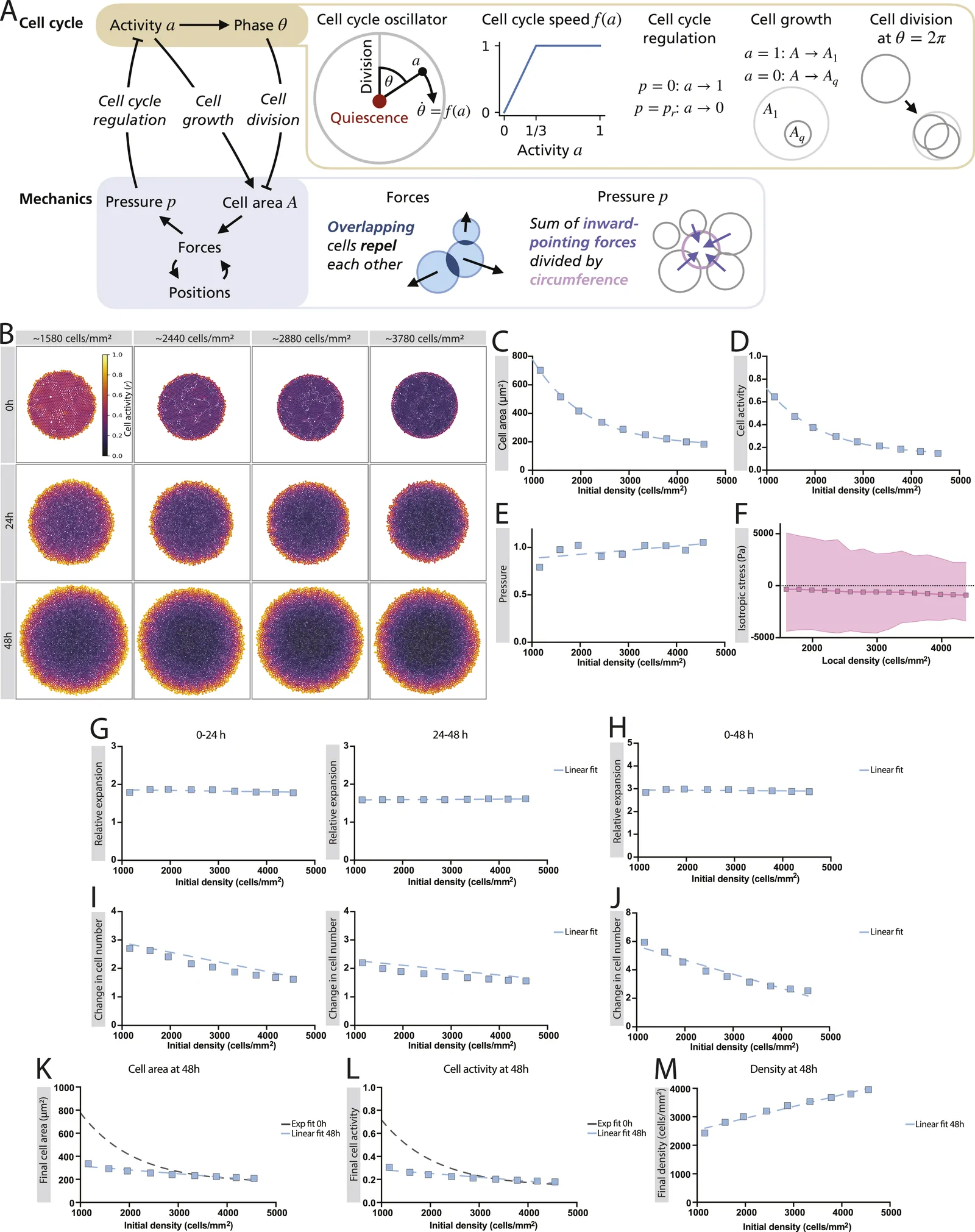
Agent-based modelling reveals mechanochemical feedback drives tissue-scale pressure regulation and density homeostasis in expanding epithelial colonies. **(A)** Cartoon of the model: Each cell contains a stylized cell cycle oscillator with phase theta and activity r. Cells divide when the phase completes a full revolution, *θ* = n*2*π (with a natural number *n*). The activity 0 < 1 <= 1 regulates the speed of the cell cycle via a function *f*(*r*) (see small panel to the right): Cell divisions happen on average every 18 h for *r* > ^1/3^ and decrease linearly with *r* for 0 < *r* < ^1/3^. **(B)** Colour maps of simulations of epithelial colonies with an initial diameter of 1 mm of varying densities ranging from ~1500 to ~3800 cells/mm^2 ^at 0, 24, and 48 h of expansion. Colour-coded according to the model read-out cell activity value from 0 to 1. **(C)** Cell area in µm²as a factor of initial density at 0 h across the density range ~1500 cells/mm^2 ^to ~4500 cells/mm^2^ (*n* = 9 colonies from model simulations). **(D)** Cell activity as a factor of initial density at 0 h across the density range ~1500 cells/mm^2 ^to ~4500 cells/mm^2^ (*n* = 9 colonies from model simulations). **(E)** Pressure as a factor of initial density at 0 h across the density range ~1500 cells/mm^2 ^to ~4500 cells/mm^2^ (*n* = 9 colonies from model simulations). **(F)** Isotropic stress (Pa) as a factor of the local density across density range of 1500 cells/mm^2^–4500 cells/mm^2^. Mean values represented with a solid connected line, whereas background points represent individual cells, shaded area represents full range of all measured cells at the given density. **(G)** Relative expansion from 0 to 24, and 24 to 48 h as a factor of initial density at 0 h across the density range ~1500 cells/mm^2 ^to ~4500 cells/mm^2^ (*n* = 9 colonies from model simulations). Relative expansion is defined as the fold change in colony area from start of expansion. Dashed line represents linear fit. **(H)** Relative expansion from 0 to 48 h as a factor of initial density at 0 h across the density range ~1500 cells/mm^2 ^to ~4500 cells/mm^2^ (*n* = 9 colonies from model simulations). Relative expansion is defined as the fold change in colony area from start of expansion. Dashed line represents linear fit. **(I)** Change in cell number from 0 to 24, and 24 to 48 h as a factor of initial density at 0 h across the density range ~1500 cells/mm^2 ^to ~4500 cells/mm^2^ (*n* = 9 colonies from model simulations). Dashed line represents linear fit. **(J)** Change in cell number from 0 to 48 h as a factor of initial density at 0 h across the density range ~1500 cells/mm^2 ^to ~4500 cells/mm^2^ (*n* = 9 colonies from model simulations). Dashed line represents linear fit. **(K)** Final cell area at 48 h as a factor of the initial density at 0 h of colonies across the density range ~1500 cells/mm^2 ^to ~4500 cells/mm^2^ (*n* = 9 colonies from model simulations). Grey dashed line represents exponential fit at 0 h and blue dashed line represents linear fit at 48 h. **(L)** Final YAP N/C ratio at 48 h as a factor of the initial density at 0 h of colonies across the density range ~1500 cells/mm^2 ^to ~4500 cells/mm^2^ (*n* = 9 colonies from model simulations). Grey dashed line represents exponential fit at 0 h and blue dashed line represents linear fit at 48 h. **(M)** Final density at 48 h as a factor of the initial density at 0 h of colonies across the density range ~1500 cells/mm^2 ^to ~4500 cells/mm^2^ (*n* = 9 colonies from model simulations). Dashed line represents linear fit.

To explore the mechanisms underlying cell number dynamics and convergence to a homeostatic state in expanding epithelial tissues post-confinement, we next turned to computational modelling. Several prior models have coupled epithelial behaviour with proliferation dynamics and demonstrated that tissues can converge to these common states, using simple forms of mechanosensitivity such as coupling tissue growth rates to local pressure or density (Basan et al, 2009; Marel et al, 2014; Bocanegra-Moreno et al, 2023; Höllring et al, 2023, 2024; Carpenter et al, 2024; Brückner & Hannezo, 2025). Although such frameworks, including models based on the Fisher–Kolmogorov equation and their mechanosensitive extensions, can exhibit convergence, they do not explicitly track the internal mechanochemical state of cells that we measured in our experiments. Thus, here we adopted a previously developed agent-based model that explicitly couples collective tissue mechanics to intracellular cell-cycle regulation and has been shown to reproduce key aspects of MDCK monolayer dynamics (Li et al, 2021, 2022, Model Suppl Info, Fig S4A–D). In this model, cells are represented as soft repulsive discs, and each cell contains a simplified cell-cycle oscillator, where proliferation and growth are governed by a cell-cycle activity variable that is down-regulated by isotropic mechanical compression, that is, pressure, experienced by cells because of crowding (Fig 3A, Model Suppl Info). This framework captures the non-instantaneous mechanochemical feedback between pressure and proliferation, conceptually analogous to experimentally observed density-dependent regulation of YAP activity and changes in cell numbers (Puliafito et al, 2012; Benham-Pyle et al, 2015; Fig 1B and C). This allows for history-dependent responses that cannot be captured by phenomenological, instantaneous coupling.

**Figure S3.**
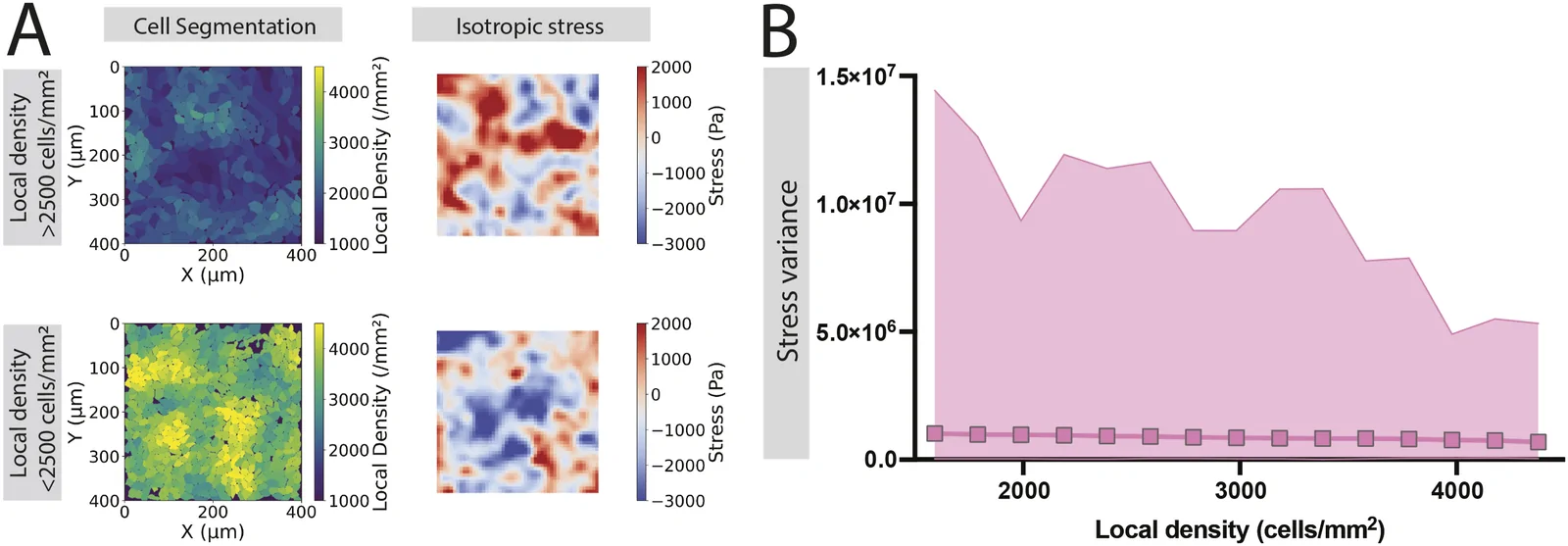
Agent-based modelling reveals mechanochemical feedback drives tissue-scale pressure regulation and density homeostasis in expanding epithelial colonies. **(A)** Representative images of the cell segmentation and isotropic stress in monolayers with a local density <2500 cells/mm^2^ and >2500 cells/mm^2 ^generated from TFM-based Bayesian force inference microscopy. Colour scale bar indicates local density (right) and stress (Pa) (left). **(B)** Stress variance as a factor of the local density across density range of 1500 cells/mm^2^–4500 cells/mm^2^. Mean values represented with a solid connected line, whilst background points represent individual cells and shaded area represents full range of all measured values at the density given by x-axis.

Because the key assumption of the model is that epithelial monolayers experience predominantly compressive stresses whose magnitude increases with cell density, we first decided to validate this experimentally. To do so, we quantified tissue-scale isotropic stresses in MDCK monolayers as a function of local cell density using monolayer stress microscopy (Tambe et al, 2013; Nier et al, 2016; Grudtsyna et al, 2025; Schoenit et al, 2025). These data showed that indeed, across an MDCK monolayer, isotropic stresses were predominantly negative indicating a compressive state and that the magnitude of compressive stresses increased monotonically with an increase in local cell density (Figs 3F and S3A and B).

**Figure S4.**
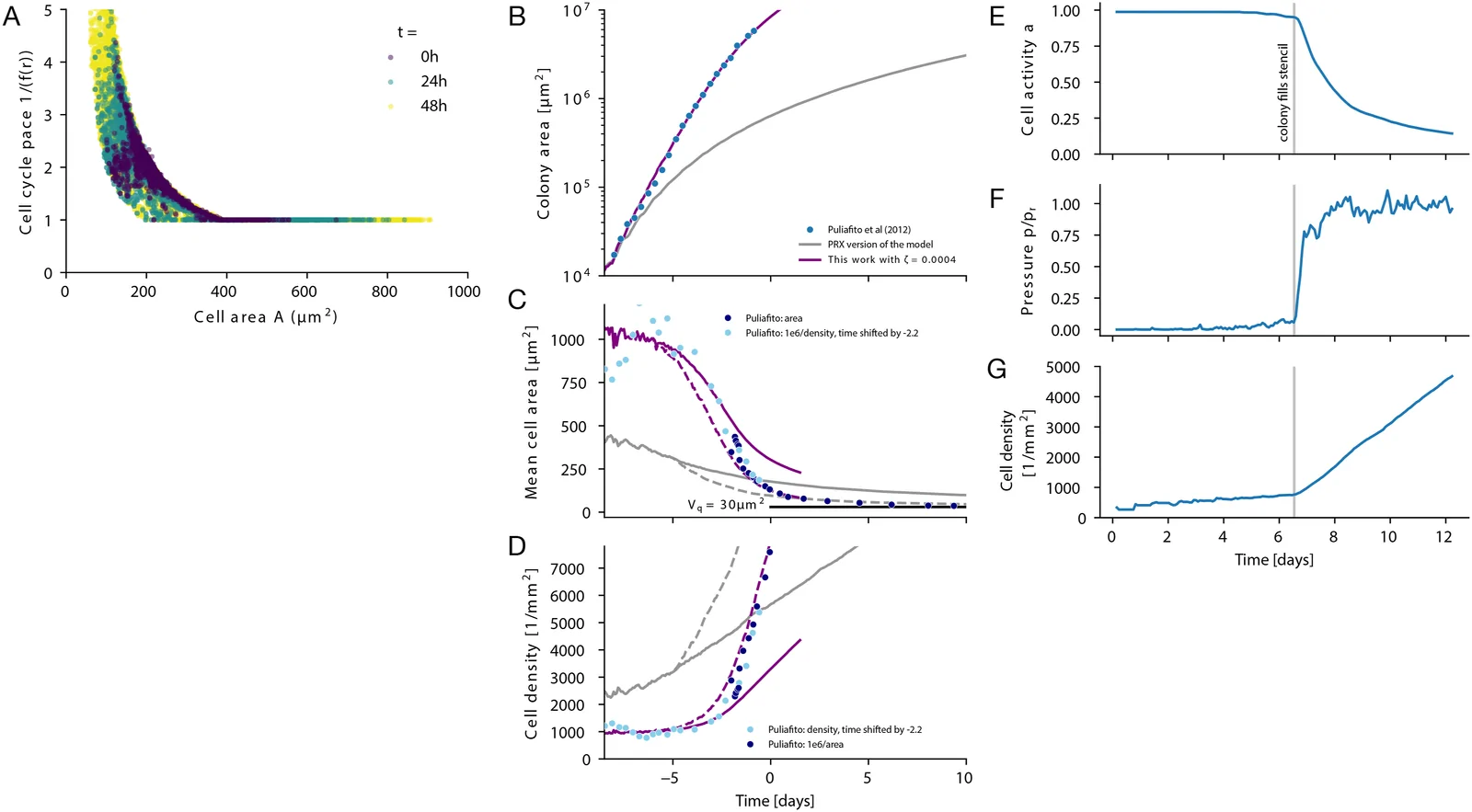
Calibration of the model. **(A)** Cell cycle pace vs. cell area. The cell cycle pace 1 *f*(*a*) is an estimator of the expected cell division time (which can exceed the duration of the simulation). A value of 1 *f*(*a*) = 1 corresponds to a division time of *τdiυ. ***(B, C, D)** Matching our simulations to experiments by Puliafito et al (2012). Total area of the colony (panel B), mean cell area (C), and cell average cell density (D) for experimental and simulation data over time. The experimental data by Puliafito et al. was extracted by hand using Webplotdigitizer [A. Rohatgi, Webplotdigitizer: Version 4.6 (2022), https://automeris.io/WebPlotDigitizer]: Colony areas were extracted from their Fig 1B, cell density from Fig 1C and the median of the cell size distribution (called cell area here) from Fig 3C. Their density data is from the inner region of the colony. Their cell area data is from an unspecified region of the colony. We found that density and median cell area could be made consistent when shifting the density data by −2.2 d. The simulations were grown from a single cell. The model labelled PRX version with parameters as reported in Li et al (2021) qualitatively, but not quantitatively, reproduces this experimental data. To achieve quantitative fit, we adjusted the simulation parameters, see Table I in Supplementary Information. The mean cell area and cell density are shown for as colony averages (solid lines) and for the centre of the colony (dashed lines). The latter is more comparable to the approach chosen by Puliafito et al. and thus yields a more fitting comparison. **(E, F, G)** A colony seeded from a single cell expands to fill a circular stencil. Cell activity a (panel E), average pressure p, normalised by the reference pressure *pr*, across the colony (F), and average cell density (G) as a function of time are plotted for simulation data of a single cell being seeded in the centre of a circular stencil confinement. The grey vertical line indicates when the colony first fills the stencil completely. Once the colony fully fills the stencil, pressure rises rapidly, leading to reduced cell activity over time. While the colony area cannot exceed the available stencilled area, the cells keep dividing and thus increase the density of the colony over time.

Next, we simulated our expansion assays in the model by allowing colonies to grow from a single cell under circular confinement until they reached set target densities similar to the density range in the experiments (∼1,200–4,500 cells/mm^2^) (Model Suppl Info, Fig S4E–G). Examination of the simulated colonies at stencil release before the onset of expansion revealed that similar to experimental results, median cell area of the colonies decreased with an increase in overall colony density (Fig 3C). Notably, although the cell activity parameter did not exhibit discrete density-dependent regimes as observed for YAP N/C in the experiments, it decreased with increasing colony density after an exponential decay that gradually approached a plateau at higher densities (Fig 3D).

To understand these results further, we next examined how the mechanical states get established in the epithelial monolayer during confinement. Simulations revealed that as cells grew and packed the stencil, they gradually accumulated internal mechanical pressure (Model Suppl Info, Fig S4E and F). This pressure continued to increase until the colony reached a stable reference pressure, defined here as the equilibrium level of isotropic compression at which further proliferation becomes suppressed (Model Suppl Info, Fig S4F). At this reference pressure, cell activity dropped sharply, reflecting the onset of confinement-induced growth inhibition (Model Suppl Info, Fig S4E). Because colonies at higher densities remained under confinement longer than colonies at lower densities, they spent more time at this reference pressure, resulting in lower activity at the time of stencil release (Model Suppl Info, Fig S4E). Despite these differences in initial density and cell activity, however, all colonies exhibited similar internal pressure distributions at the moment of release, although the pressure was slightly increased with increasing density, which led to comparable up-regulation of activity during early expansion (Fig 3D and E).

Upon reaching the target density, the stencils were released, and the simulated colonies were allowed to expand for 48 h. Results on overall expansion size showed that again similar to experiments, all colonies expanded to similar final size independent of initial density and the starting cell activity parameter. Together with our experimental result, these results indicate that overall epithelial expansion post-confinement is independent of density and mechanochemical state at confinement (Fig 3B and F–I).

After stencil release, simulations showed that initial differences in activity and cell size progressively diminished over time. Across the full range of initial densities, both cell area and cell activity evolved from being strongly dependent on initial density at the onset of expansion to exhibiting only a weak dependence by 48 h (Fig 3J and K). Colonies initiated at lower densities exhibited higher proliferation rates during the early phase of expansion, whereas colonies initiated at higher densities increased their proliferation more gradually, such that proliferation rates converged by 48 h (Fig 3H and I). Because of these coordinated dynamics, colonies expanded by comparable amounts and progressively approached a similar final density across the full range of starting conditions by 48 h just as in our experimental observations (Figs 3L and M and S4G).

Together, these results demonstrate that pressure-mediated feedback on proliferation and growth is sufficient to qualitatively reproduce the density-independent expansion and convergence of cell size and density observed during epithelial tissue spreading. This minimal model thus offers a mechanistic framework for how local mechanical compression can regulate global tissue behaviour, enabling robust coordination of homeostasis during dynamic epithelial remodelling.

### Reduction of internal pressure during confinement results in density-dependent changes in tissue expansion

**Figure 4.**
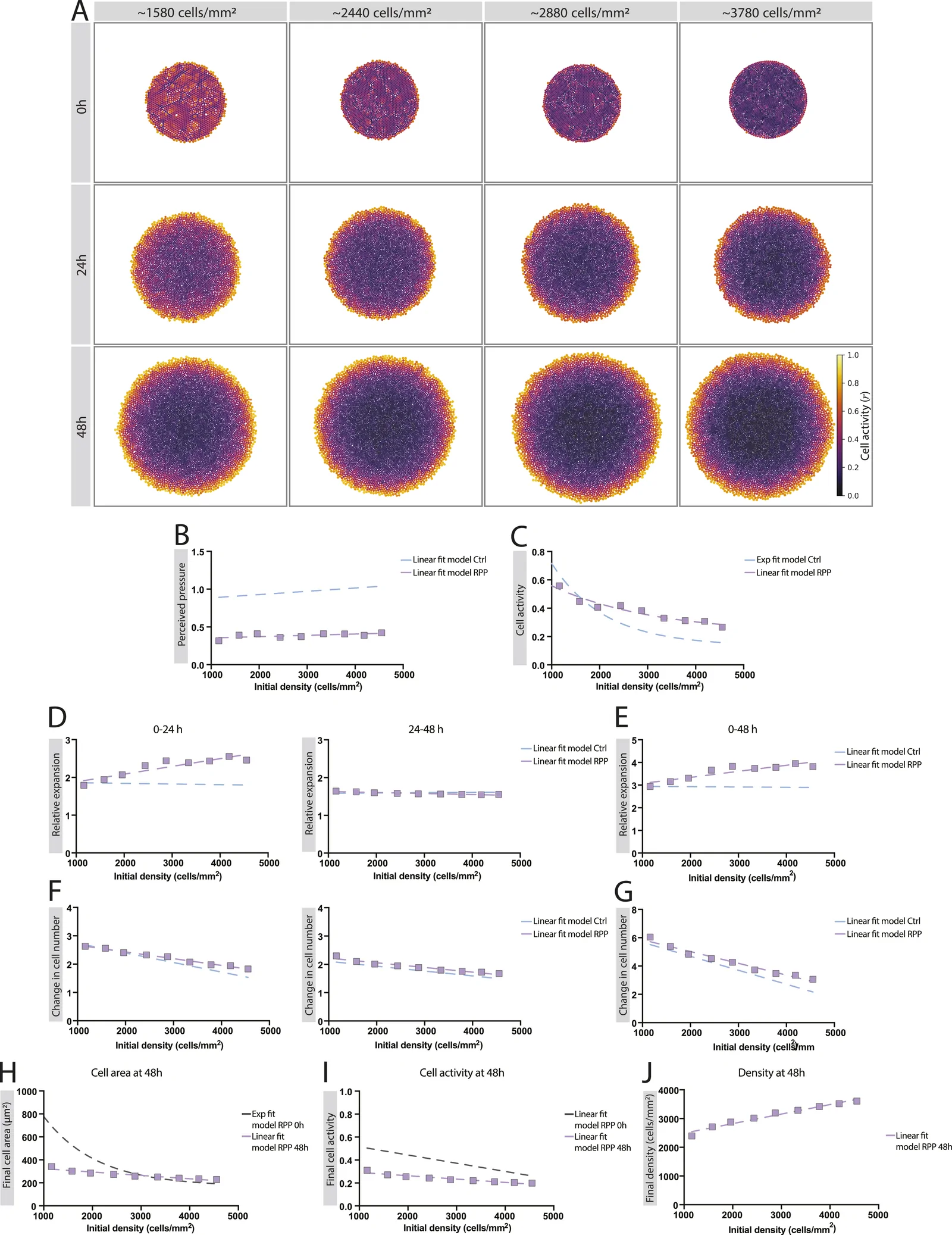
Reduction of internal pressure during confinement results in density-dependent changes in tissue expansion. **(A)** Colour maps of RPP condition simulations of epithelial colonies with an initial diameter of 1 mm of varying initial densities ranging from ~1500 to ~3800 cells/mm^2 ^at initiation of simulation (0 h) and after 24 and 48 h of expansion. Colour-coded according to the model read-out cell activity value from 0 to 1. **(B)** Perceived pressure as a factor of initial density at initiation of experiments (0 h) across the density range ~1500 cells/mm^2 ^to ~4500 cells/mm^2^ for control and reduced perceived pressure (RPP) colonies (*n* = 9 colonies from model simulations). Purple dashed line representing the linear fit of RPP colonies and blue dashed line represents the linear fit of Ctrl colonies. **(C)** Cell activity as a factor of initial density at initiation of experiments (0 h) across the density range ~1500 cells/mm^2 ^to ~4500 cells/mm^2^ for control and reduced perceived pressure (RPP) colonies (*n* = 9 colonies from model simulations). Purple dashed line representing the non-linear fit of RPP colonies and blue dashed line represents the non-linear fit of Ctrl colonies. **(D)** Relative expansion from 0 to 24, and 24 to 48 h as a factor of initial density at 0 h across the density range ~1500 cells/mm^2 ^to ~4500 cells/mm^2^ (*n* = 9 colonies from model simulations). Relative expansion is defined as the fold change in colony area from start of expansion. Dashed line represents linear fit. **(E)** Relative expansion from 0 to 48 h as a factor of initial density at 0 h across the density range ~1500 cells/mm^2 ^to ~4500 cells/mm^2^ (*n* = 9 colonies from model simulations). Relative expansion is defined as the fold change in colony area from start of expansion. Purple dashed line representing the linear fit of RPP colonies and blue dashed line represents the linear fit of Ctrl colonies. **(F)** Change in cell number from 0 to 24, and 24 to 48 h as a factor of initial density at 0 h across the density range ~1500 cells/mm^2 ^to ~4500 cells/mm^2^ (*n* = 9 colonies from model simulations). Purple dashed line representing the linear fit of RPP colonies and blue dashed line represents the linear fit of Ctrl colonies. **(G)** Change in cell number from 0 to 48 h as a factor of initial density at 0 h across the density range ~1500 cells/mm^2 ^to ~4500 cells/mm^2^ (*n* = 9 colonies from model simulations). Purple dashed line representing the linear fit of RPP colonies and blue dashed line represents the linear fit of Ctrl colonies. **(H)** Final cell area at 48 h as a factor of the initial density at 0 h of RPP colonies across the density range ~1500 cells/mm^2 ^to ~4500 cells/mm^2^ (*n* = 9 colonies from model simulations). Grey dashed line represents exponential fit at 0 h and purple dashed line represents linear fit at 48 h. **(I)** Final YAP N/C ratio at 48 h as a factor of the initial density at 0 h of RPP colonies across the density range ~1500 cells/mm^2 ^to ~4500 cells/mm^2^ (*n* = 9 colonies from model simulations). Grey dashed line represents linear fit at 0 h and purple dashed line represents linear fit at 48 h. **(J)** Final density at 48 h as a factor of the initial density at 0 h of RPP colonies across the density range ~1500 cells/mm^2 ^to ~4500 cells/mm^2^ (*n* = 9 colonies from model simulations). Dashed line represents linear fit.

Results from the model above suggest that pressure builds up during confinement and the cell’s ability to sense this pressure plays a central role in regulating proliferation and restoring homeostasis after expansion. Because our model tracks cell cycle–dependent activity, it also allows us to examine how mechanical history influences subsequent growth dynamics. As such, we next asked whether transiently altering how cells perceive pressure could shift their expansion behaviour or affect re-establishment of homeostasis. To this end, we introduced a reduced perceived pressure (RPP) condition in the model (Fig 4A).

**Figure S5.**
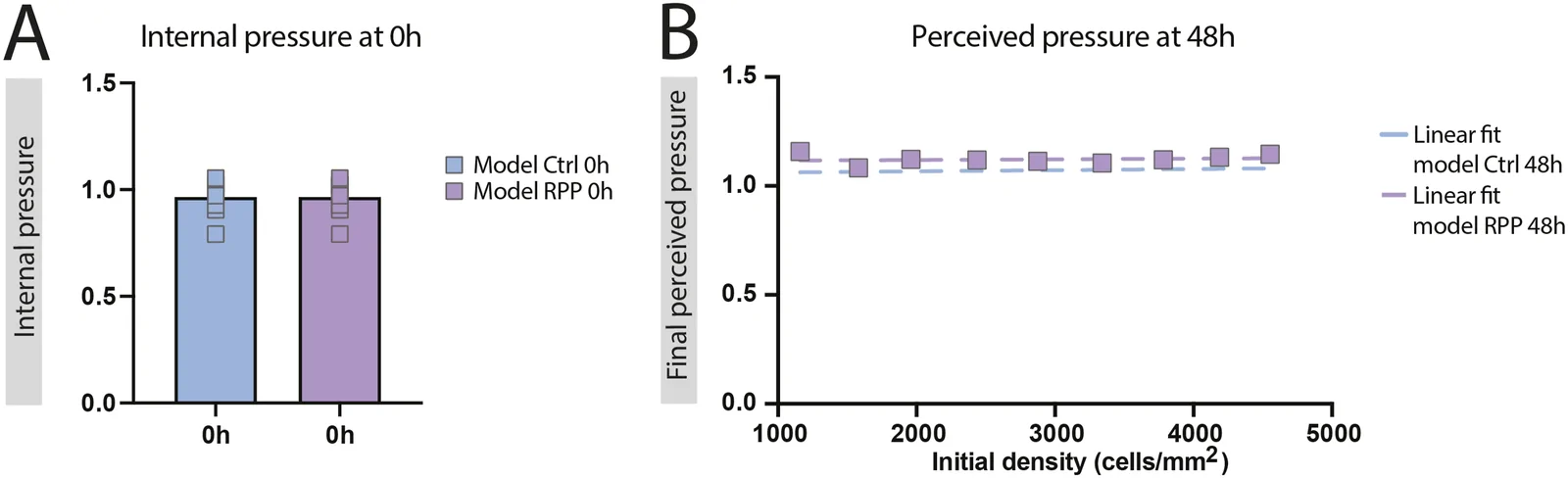
Reduction of internal pressure during confinement results in density-dependent tissue expansion. **(A)** Internal pressure in model Ctrl and RPP colonies at initiation of experiments (0 h) (*n* = 9 Ctrl and 9 RPP colonies from model simulation runs). **(B)** Final perceived pressure in model Ctrl and RPP colonies after 48 hours of expansion as a function of the initial density (*n* = 9 Ctrl and 9 RPP colonies from model simulation runs). Dashed line in purple represents linear fit of RPP colonies and dashed line in blue represents linear fit of Ctrl colonies at 48 h.

Here, we selectively dampened the mechanical signal sensed by cells, without altering the actual tissue-level pressure (Fig S5A). Specifically, we lowered the perceived pressure by 60% for a 3-h period immediately before stencil release across the full range of initial densities, while disabling cell growth and division during this time to isolate the effect (Fig 4B). Surprisingly, this brief reduction in perceived pressure had a strong, density-dependent effect on cell activity (Fig 4C). Colonies initiated at lower densities, where activity was near-maximal, showed little response to RPP, whereas colonies initiated at higher densities exhibited a marked increase in activity relative to the control (Fig 4C). This reflects the fact that colonies experiencing stronger confinement-induced suppression before release respond more strongly to transient relief of perceived pressure, restoring activity that would otherwise remain low. At the time of confinement release, RPP-treated colonies exhibited elevated activity compared with control colonies of comparable density, demonstrating that cellular responses to pressure depend on prior mechanical history rather than initial density alone. After stencil release, we reinstated cell growth and division and allowed colonies to expand freely for 48 h (Fig 4A). During this phase, the RPP condition was gradually reversed and perceived pressure sensing recovered exponentially towards the model-defined reference pressure (Fig S5B). Despite this restoration, the early colony density-dependent change in activity induced by RPP had lasting consequences; during the first 24 h, the rate of expansion increased with initial density, driven by a sustained increase in proliferation (Fig 4D–G). Strikingly, this enhanced expansion in RPP colonies with higher initial density acted as a compensatory response to the early surge in proliferation, allowing the tissue to regulate its density and converge with lower density colonies showing less expansion over time. As a result, despite starting from distinct mechanochemical states, all colonies ultimately started converging to similar final cell densities by 48 h with cell activity and average cell sizes also equilibrated across the density range conditions after 48 h of expansion (Fig 4H–J).

Together, these results show that transient modulation of pressure sensing during confinement alters early expansion dynamics in a density-dependent manner. Colonies subjected to RPP exhibited enhanced early expansion at higher densities, whereas all conditions ultimately converged to a common final density. Thus, transient mechanical perturbations reshape expansion trajectories without disrupting robust restoration of tissue homeostasis.

### Transient mechanical resetting during confinement selectively elevates YAP activity at higher densities

**Figure S6.**
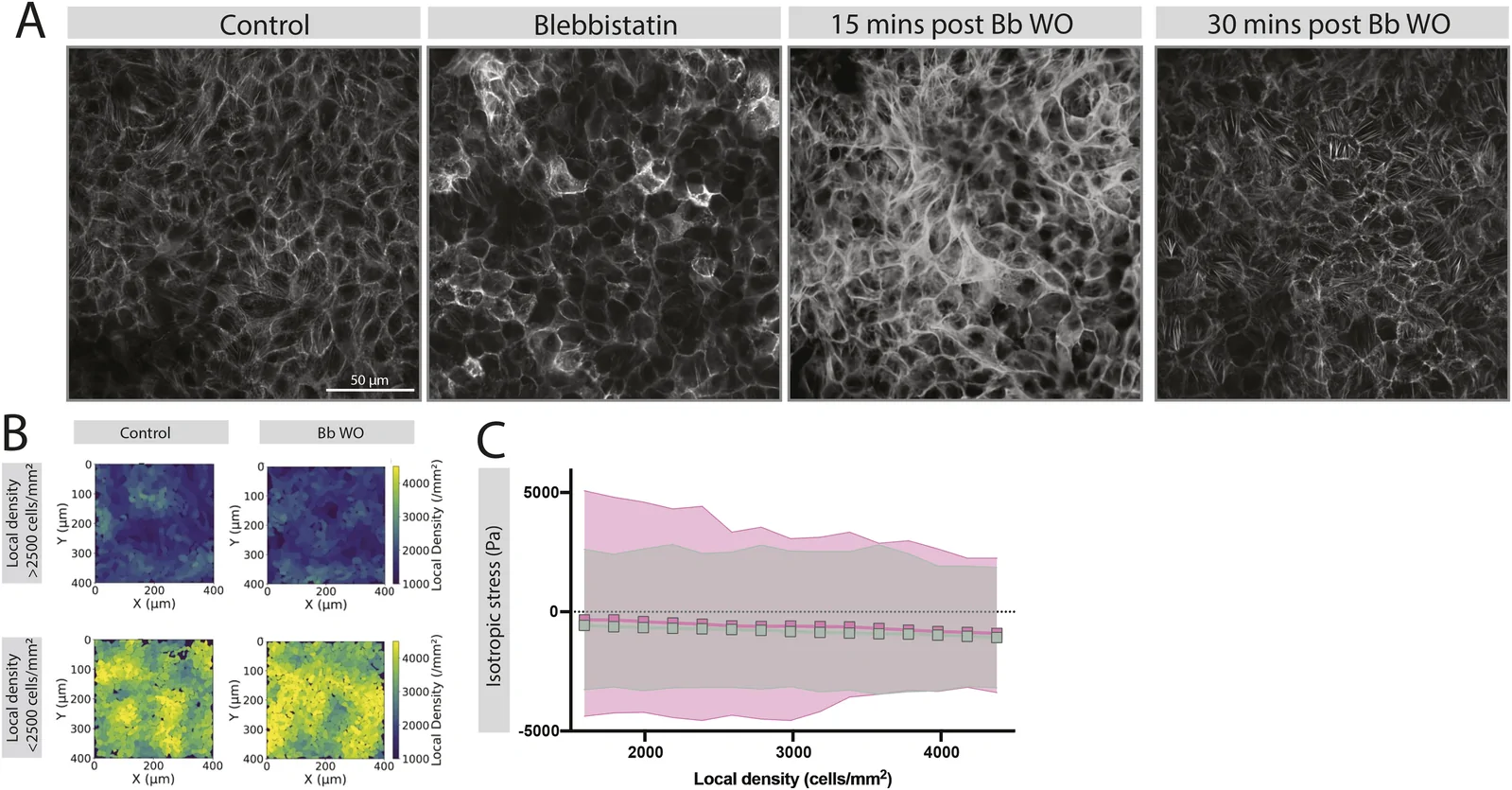
Transient mechanical resetting during confinement selectively elevates YAP activity at higher densities. **(A)** Representative images of actin in MDCK cells of following conditions: no treatment (control), blebbistatin treatment, blebbistatin wash out 15 mins post WO and blebbistatin WO 30 mins post WO: Scale bar 50 µm. **(B)** Representative images of the cell segmentation and isotropic stress in control and Bb WO monolayers with a local density <2500 cells/mm^2^ and >2500 cells/mm^2 ^generated from TFM-based Bayesian force inference microscopy. Colour scale bar indicates local density (right) and stress (Pa) (left). **(C)** Isotropic stress (Pa) as a factor of the local density across density range of 1500 cells/mm^2^–4500 cells/mm^2 ^in control and Bb WO monolayers. Mean values represented with a solid connected line, whilst background points represent individual cells, shaded area represents full range of all measured cells at the given density.

To experimentally test the model’s prediction that transient reductions in perceived mechanical pressure can modulate cell numbers in a density-dependent manner, we sought an approach to mimic the RPP condition used in simulations. In the model, RPP temporarily decouples the isotropic stresses experienced by cells under confinement from the mechanotransduction signal that regulates their activity, revealing a latent capacity for high-density tissues to re-activate and expand. Because cells in epithelial tissues sense pressure indirectly through local mechanical stresses, such as cortical tension and junctional forces, we used a blebbistatin (Bb) washout (Bb WO) approach. Bb, a myosin II inhibitor that reduces actomyosin contractility and lowers cortical tension in epithelial cells, has been shown to decrease cell-generated stresses and impair mechanosensing in MDCK epithelial monolayers (Cai et al, 2006; Hung et al, 2016; Chen et al, 2018; Di Meglio et al, 2022; Thiagarajan et al, 2022; Yang et al, 2022; Schoenit et al, 2025). Although Bb affects multiple aspects of cell physiology, its most direct and well-established effect in this context is the transient reduction of actomyosin-generated mechanical stresses (Hung et al, 2016; Chen et al, 2018; Ajeti et al, 2019; Schoenit et al, 2025). To verify the effectiveness and transient nature of Bb WO, we assessed cytoskeletal organization and tissue-scale mechanics in confluent MDCK epithelial monolayers after Bb treatment and Bb WO. As expected, actin fibres were no longer visible across the epithelial monolayer consistent with loss of cortical actin and isotropic stresses after 1 h of 50 μM Bb treatment (Fig S6A). We then washed out the Bb and added fresh media and could observe recovery of stress fibres within the first 15 min (Fig S6A). By 30 min of washout, actin fibres had completely recovered to levels comparable to untreated controls (Fig S6A). To then verify functional recovery, we performed monolayer stress microscopy and found that 30 min after washout was sufficient for recovery of mean isotropic stress to near-control levels, while maintaining the normal density-dependent scaling of compressive stress across the monolayer (Fig S6B and C). Notably, Bb WO also resulted in a significant reduction in stress heterogeneity across the monolayer, indicating a more mechanically homogeneous tissue state (Fig S6C). Together with previous studies showing that Bb treatment reduces isotropic stresses in MDCK monolayers (Ajeti et al, 2019; Schoenit et al, 2025), these results demonstrate that transient Bb treatment followed by washout induces a uniform mechanical reset at the tissue scale, without altering epithelial integrity or cell density.

**Figure 5.**
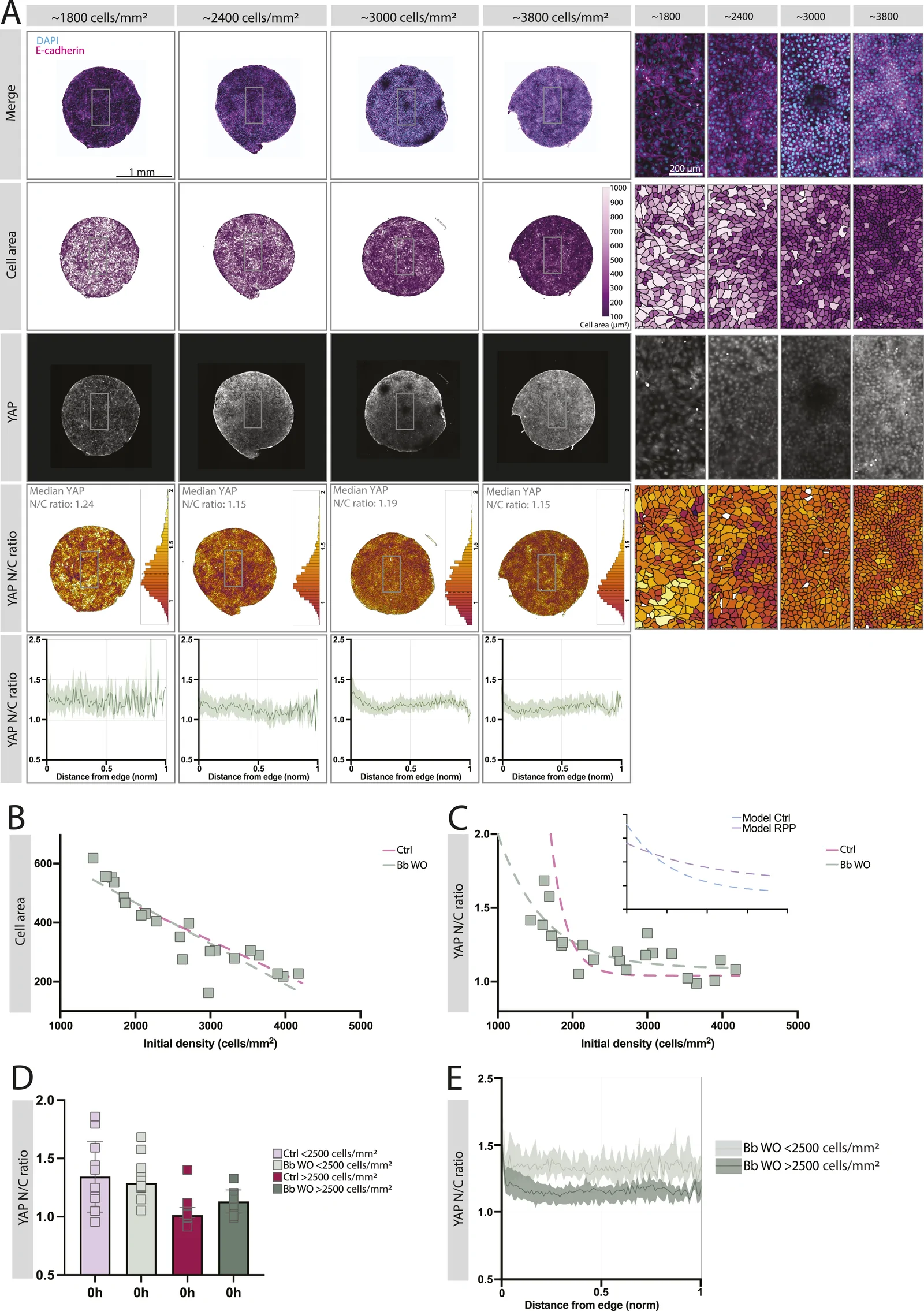
Transient mechanical resetting during confinement selectively elevates YAP activity at higher densities. **(A)** Representative images of DAPI/E-cadherin merge, cell area colour maps, YAP channel, and YAP N/C colour maps of colonies of varying densities ranging from ~1800 to ~3800 cells/mm^2 ^after Bb WO treatment. Images taken after removal of stencil, color-coded for cell area and YAP N/C ratio. Representative histograms display the population spread for YAP N/C ratios and colour scale from 0.8 to 2. The dashed lines indicate the median YAP N/C ratio for the colony. Radial spatial plots displaying median YAP N/C ratio of example colonies. Thicker line represents median YAP across colony, with shaded error bars representing interquartile range (IQR) of these. X-axis represents the distance from edge to centre, which has been normalized to arbitrary values. Scale bar (whole colony images). Scale bar (zoom in images) 200 µm. **(B)** Cell area in µm² as a factor of initial density at 0 h across the density range ~1400 cells/mm^2^ to ~4200 cells/mm^2^ after Bb WO treatment (*n* = 22 colonies from at least six individual experiments). Green dashed line represents linear fit of Bb WO colonies and pink dashed line represents linear fit of Ctrl colonies. **(C)** YAP N/C ratio as a factor of initial density at 0 h across the density range ~1700 cells/mm^2 ^to ~4500 cells/mm^2 ^after Bb WO treatment. (*n* = 22 colonies from at least six individual experiments). Dashed line in green represents non-linear fit of Bb WO colonies, dashed line in pink represents non-linear fit of Ctrl colonies. Inset showing non-linear fits of model ctrl (blue) and model RPP (purple). **(D)** YAP N/C ratio of control and Bb WO colonies <2500 and >2500 cells/mm^2 ^imaged and measured after removal of stencil (*n* = 11 <2500 cells/mm^2^ and 13 >2500 cells/mm^2^ colonies from at least eight individual experiments, and 10 LD Bb <2500 cells/mm^2^ and 12 Bb WO >2500 cells/mm^2^ colonies from at least six individual experiments). **(E)** Radial spatial plots displaying median YAP N/C ratio of Bb WO colonies, divided into colonies with a density <2500 cells/mm^2^ and >2500 cells/mm^2^. Thicker line represents median YAP ratio across colony, with shaded error bars representing interquartile range (IQR) of these. X-axis represents the distance from edge to centre, which has been normalized to arbitrary values.

After confirming the effect of Bb WO, epithelial monolayers were grown in PDMS stencils across the density range just as before and treated with 50 μM Bb for 1 h upon reaching target densities, washed out, and then allowed to recover for 30 min before stencil release. Colonies were then fixed at the time of stencil removal and analysed for cell area and YAP N/C to quantify the mechanochemical state before the onset of expansion (Fig 1A). Analysis of cell area across all colonies showed that median cell area in Bb WO colonies still decreased monotonically with increasing initial density, similar to control colonies in Fig 1 (Fig 5A and B). YAP N/C in Bb WO colonies also showed density dependence in Bb WO colonies though with differences compared with control colonies (Fig 5C). Bb WO led to a selective increase in YAP N/C at higher densities while having a minimal effect at lower densities, resulting in a flatter YAP N/C–density relationship compared with the control (Fig 5A and C). This density-dependent upward shift in YAP N/C closely mirrored the change in cell activity predicted by the model under RPP conditions. To directly compare the effect across densities with controls in Fig 1, colonies were grouped by initial density just as before. In colonies <2,500 cells/mm^2^, YAP N/C values in Bb WO conditions were not significantly different from those observed in control colonies. In contrast, Bb WO colonies >2,500 cells/mm^2^ exhibited a significant increase in YAP N/C (Fig 5A and D). Spatial analysis further showed that this increase in Bb WO colonies >2,500 cells/mm^2^ reflected a population-wide shift towards more nuclear YAP, with high YAP N/C cells homogeneously distributed throughout the colony rather than restricted to specific regions or the colony edge (Fig 5E).

Together, these results show that Bb WO acts as a transient mechanical reset of epithelial colonies during confinement. This reset selectively alters the mechanochemical state of higher density colonies, elevating YAP activity in a manner that closely mirrors the RPP condition in the model.

### Resetting stresses during confinement leads to changes in density-dependent tissue expansion

**Figure 6.**
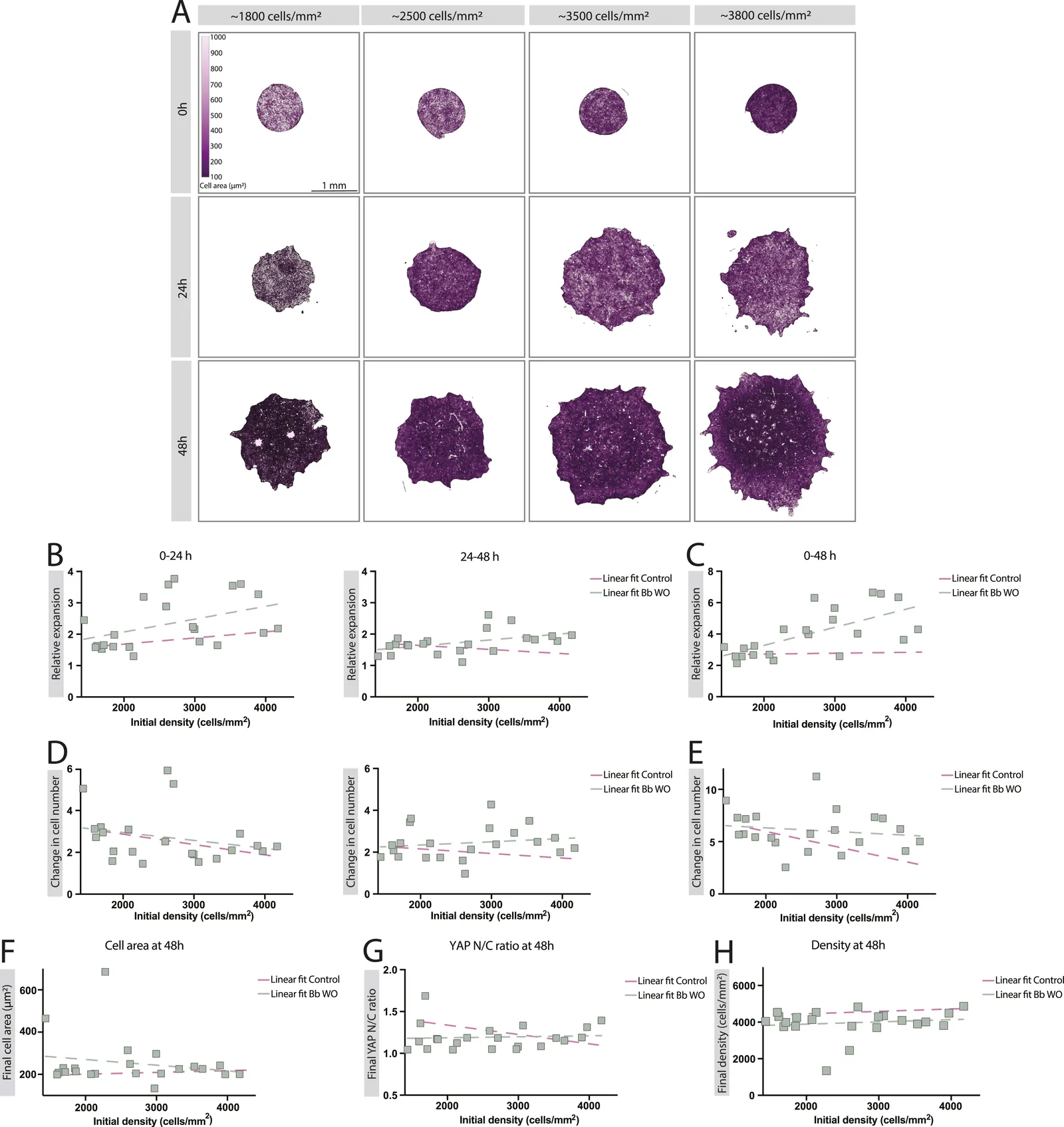
Resetting stresses during confinement leads to changes in density-dependent tissue expansion. **(A)** Cell area colour maps of representative epithelial colonies with an initial diameter of 1.5 mm of varying densities ranging from ~1800 to ~3800 cells/mm^2 ^after Bb WO treatment. Images acquired after 0, 24, and 48 h of expansion, segmented, and colour-coded according to cell area and with cell membrane outlines drawn as overlay. Scale bar, 1 mm. **(B)** Relative expansion from 0 to 24, and 24 to 48 h as a factor of initial density at 0 h across the density range ~1400 cells/mm^2 ^to ~4500 cells/mm^2^ after Bb WO treatment (*n* = 22 colonies from at least six individual experiments). Dashed line in green represents linear fit of Bb WO colonies, dashed line in pink represents linear fit of control colonies. **(C)** Relative expansion from 0 to 48 h as a factor of initial density at 0 h across the density range ~1400 cells/mm^2 ^to ~4500 cells/mm^2^ Bb WO treatment (*n* = 22 colonies from at least six individual experiments). Dashed line in green represents linear fit of Bb WO colonies, dashed line in pink represents linear fit of control colonies. **(D)** Change in cell number from 0 to 24, and 24 to 48 h as a factor of initial density at 0h across the density range ~1400 cells/mm^2 ^to ~4500 cells/mm^2^ Bb WO treatment (*n* = 22 colonies from at least six individual experiments). Dashed line in green represents linear fit of Bb WO colonies, dashed line in pink represents linear fit of control colonies. **(E)** Change in cell number from 0 to 48 h as a factor of initial density at 0 h across the density range ~1400 cells/mm^2 ^to ~4500 cells/mm^2^ Bb WO treatment (*n* = 22 colonies from at least six individual experiments). Dashed line in green represents linear fit of Bb WO colonies, dashed line in pink represents linear fit of control colonies. **(F)** Final cell area at 48 h as a factor of the initial density at 0 h of colonies across the density range ~1400 cells/mm^2 ^to ~4500 cells/mm^2^ after Bb WO treatment (*n* = 22 colonies from at least six individual experiments). Dashed line in green represents linear fit of Bb WO colonies, dashed line in pink represents linear fit of control colonies. **(G)** Final YAP N/C ratio at 48 h as a factor of the initial density at 0 h of colonies across the density range ~1400 cells/mm^2 ^to ~4500 cells/mm^2^ after Bb WO treatment (*n* = 22 colonies from at least six individual experiments). Dashed line in green represents linear fit of Bb WO colonies, dashed line in pink represents linear fit of control colonies. **(H)** Final density at 48 h as a factor of the initial density at 0 h of colonies across the density range ~1400 cells/mm^2 ^to ~4500 cells/mm^2^ after Bb WO treatment (*n* = 22 colonies from at least six individual experiments). Dashed line in green represents linear fit of Bb WO colonies, dashed line in pink represents linear fit of control colonies.

We next allowed Bb WO colonies to expand for 24 and 48 h after stencil release before fixation and analysis of expansion and changes in cell number (Fig 6A). Compared with control colonies, Bb WO colonies exhibited marked differences in expansion at both time points (Fig 6B–C). During the first 24 h after release, Bb WO colonies expanded more than controls across the full range of initial densities, with this effect being most pronounced in colonies initiated at higher densities (Fig 6B). During the subsequent 24 h, this enhancement in expansion was no longer apparent at lower initial densities, whereas colonies initiated at higher densities maintained a modestly elevated expansion rate relative to controls (Fig 6B and C). As a result, Bb WO colonies displayed a clear initial density dependence in cumulative expansion over 48 h, with higher density colonies expanding significantly more than lower density Bb WO colonies and control colonies (Fig 6C). This enhanced expansion in higher density Bb WO colonies was accompanied by a selective, density-dependent increase in cell number (Fig 6D and E). Consequently, net changes in cell number in Bb WO colonies became largely independent of initial density over the 48-h expansion period, in contrast to the density dependence observed in control colonies (Fig 6E).

**Figure S7.**
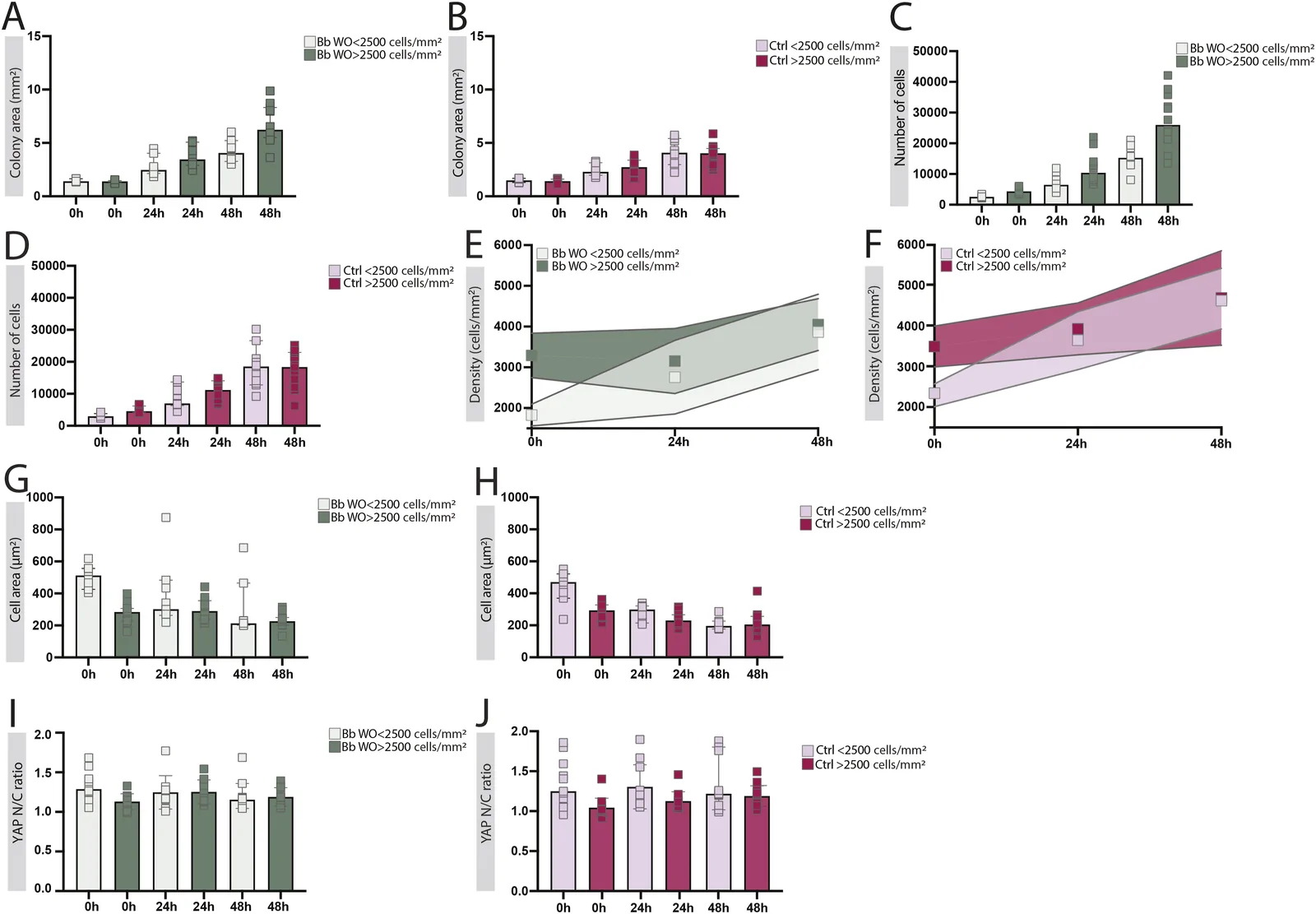
Resetting stresses during confinement leads to changes in density-dependent tissue expansion. **(A)** Colony area in Bb WO colonies <2500 and >2500 cells/mm^2 ^after 0, 24 and 48 h of expansion (*n *= 10 <2500 and 12 >2500 cells/mm^2^ colonies from at least six individual experiments). **(B)** Colony area in Ctrl colonies <2500 and >2500 cells/mm^2 ^after 0, 24 and 48 h of expansion (*n *= 11 <2500 and 13 >2500 cells/mm^2^ colonies from at least eight individual experiments). **(C)** Number of cells in Bb WO colonies <2500 and >2500 cells/mm^2 ^after 0, 24 and 48 h of expansion (*n *= 10 <2500 and 12 >2500 cells/mm^2^ colonies from at least six individual experiments). **(D)** Number of cells in Ctrl colonies <2500 and >2500 cells/mm^2 ^after 0, 24 and 48 h of expansion (*n *= 11 <2500 and 13 >2500 cells/mm^2 ^colonies from at least eight individual experiments). **(E)** Density over time in Bb WO colonies <2500 and >2500 cells/mm^2 ^after 0, 24 and 48 h of expansion (*n *= 10 <2500 and 12 >2500 cells/mm^2^ colonies from at least six individual experiments). **(F)** Density over time in Ctrl colonies <2500 and >2500 cells/mm^2 ^after 0, 24 and 48 h of expansion (*n *= 11 <2500 and 13 >2500 cells/mm^2^ colonies from at least eight individual experiments). **(G)** Cell area in Bb WO colonies <2500 and >2500 cells/mm^2 ^after 0, 24 and 48 h of expansion (*n *= 10 <2500 and 12 >2500 cells/mm^2^ colonies from at least six individual experiments). **(H)** Cell area in Ctrl colonies <2500 and >2500 cells/mm^2 ^after 0, 24 and 48 h of expansion (*n *= 11 <2500 and 13 >2500 cells/mm^2^ colonies from at least eight individual experiments). **(I)** YAP N/C ratio in Bb WO colonies <2500 and >2500 cells/mm^2 ^after 0, 24 and 48 h of expansion (*n *= 10 <2500 and 12 >2500 cells/mm^2^colonies from at least six individual experiments). **(J)** YAP N/C ratio in Ctrl colonies <2500 and >2500 cells/mm^2 ^after 0, 24 and 48 h of expansion (*n *= 11 <2500 and 13 >2500 cells/mm^2^ colonies from at least eight individual experiments).

Despite these altered expansion trajectories, analysis across the full range of initial densities revealed that Bb WO colonies converged to the final density and YAP N/C by 48 h, indicating preservation of density homeostasis (Fig 6F and G). Average cell area also converged across conditions, confirming that differences in final colony size reflected differences in cell number rather than sustained changes in cell size (Fig 6H). To further facilitate direct comparison with control experiments and all model predictions, colonies were again grouped based on the YAP N/C–defined density regimes in control colonies. In this binned analysis, Bb WO colonies with initial densities >2,500 cells/mm^2^ exhibited a significantly greater increase in colony area and cell number compared with all other conditions, whereas colonies <2,500 cells/mm^2^ showed no difference (Fig S7A–D). Across all conditions, binned analyses confirmed convergence of final density, cell area, and YAP activity, consistent with the experimentally observed and model-predicted approach to a common homeostatic state (Fig S7E–J).

Together, these results demonstrate that transient reduction of crowding-induced pressure sensing during confinement selectively alters density-dependent population growth in high-density tissues. This confirms the model’s prediction that epithelial expansion is regulated by time-integrated mechanical stress and mechanical memory, rather than by instantaneous density alone.

## Discussion

In this study, we provide an experimental demonstration and theoretical rationalization of a reversible, history-dependent pressure-sensing mechanism that links molecular activity, cell-cycle dynamics, and collective tissue expansion. In agreement with earlier studies, epithelial monolayers starting from a wide range of initial densities expand to similar final sizes and densities, revealing an emergent capacity for tissue-scale self-regulation. Here, we show that this convergence occurs despite pronounced differences in initial YAP activity, cell size, and cell number dynamics, indicating that expansion is governed by dynamically regulated collective behaviour rather than initial conditions alone. A mechanochemical model that incorporates pressure-mediated feedback on cell-cycle activity recapitulates these dynamics. In the model, pressure builds during confinement, modulates progression through the cell cycle, and thereby regulates cell number accumulation, leading to convergence in density. Transient perturbation of this pressure-mediated feedback, either in silico by reducing perceived pressure or in vitro via Bb WO , selectively restores cell-cycle activity in higher density tissues while preserving the final homeostatic state. Together, these results support a framework in which the internal state of cells integrates local mechanical stress and crowding over time, coordinating collective growth to robustly maintain epithelial homeostasis.

Previous work has shown that epithelial tissues can maintain functional integrity despite local heterogeneity in signalling and morphology, and that confinement can modulate growth through mechanical feedback (Serra-Picamal et al, 2012; Marel et al, 2014; Streichan et al, 2014; Puliafito et al, 2017; Heinrich et al, 2020; Di Meglio et al, 2022; Devany et al, 2023; Höllring et al, 2023; Kawaue et al, 2023). Such behaviour is well captured by continuum and mean-field frameworks, including Fisher–Kolmogorov-type equations and their mechanosensitive extensions, in which growth rates decrease with increasing density or pressure. These models also successfully explain how tissues can average out local variability and converge towards a shared target state. However, most of these approaches assume an instantaneous coupling between mechanical signals and cellular responses. Our findings indicate that this assumption is insufficient to capture the full range of tissue-scale behaviours observed during expansion. Instead, epithelial cells retain an internal dynamic state, here reflected in cell-cycle activity, that integrates mechanical inputs over time and causally modulates collective expansion behaviour. As a result, transient perturbations of pressure sensing can produce pronounced, density-selective differences in early expansion dynamics even when boundary conditions and initial densities are identical. Importantly, despite these divergent early trajectories, tissue-scale homeostasis remains robust, with coordinated expansion restoring pressure balance and enabling convergence to a common final density.

Central to this process appears to be a set of mechanosensitive regulators of cell activity, including, but not limited to, the transcriptional co-activator YAP. In our system, YAP activity was spatially uniform and dynamically modulated by both initial density and changes in mechanical stress. Similar roles have been reported for other mechanosensitive regulators, including β-catenin, Piezo1, and cyclin D1, in controlling stretch- or density-dependent cell-cycle progression (Coste et al, 2010; Benham-Pyle et al, 2015; Gudipaty et al, 2017; Devany et al, 2023). These factors may act in parallel or downstream of shared mechanical signals, forming a distributed control system that links tissue-scale mechanics to cell-cycle regulation. Such a separation between mechanical sensing and downstream execution may enable flexible yet robust control of growth and division under dynamic conditions. Consistent with this view, the selective restoration of activity in higher density colonies after transient stress reduction supports the existence of an integrated, feedback-driven mechanism governing collective tissue growth.

Our modelling and Bb WO experiments suggest that total pressure, emerging from confinement and cumulative mechanical stress, acts as a mechanical signal that is sensed by cells and incorporated into their internal regulatory state. Colonies that experienced prolonged high pressure exhibited suppressed cell-cycle activity and reduced cell number accumulation, whereas transient mechanical relaxation reversed this suppression and restored growth capacity, without altering the final density achieved. This points to a feedback mechanism in which cells integrate mechanical history through their internal state to regulate growth and division dynamics, resembling checkpoint-like behaviour. Similar mechanically regulated checkpoints have been described in other systems, including tension-sensitive G2 arrest via E-cadherin–Wee1 signalling, as well as β-catenin– or cyclin D1–mediated responses to crowding, suggesting that such feedback loops may be a general feature of mechanically regulated tissues (Benham-Pyle et al, 2015; Di Meglio et al, 2022; Donker et al, 2022; Devany et al, 2023). Together, these findings extend existing frameworks of mechanical control by explicitly incorporating cellular memory and internal state.

We note that recent models represent important advances in mechanosensitive tissue modelling (Höllring et al, 2023, 2024). Whether such frameworks can reproduce the full set of history-dependent responses revealed by transient mechanical resetting remains to be tested. In addition, the experimental findings in this work derive only from mechanical perturbations via Bb treatment and Bb WO, and an orthogonal approach examining other mechanical pathways such as activation of Piezo1, other mechanosensitive channels, or other more specific perturbations will extend these observations in future studies. In this context, our study provides an experimental benchmark and theoretical rationale for a reversible, history-dependent pressure-sensing mechanism that links molecular activity, cell-cycle dynamics, and collective tissue expansion.

## Materials and Methods

### Cell culture

E-cadherin-RFP–expressing MDCK gII cells were cultured in DMEM low glucose (31885023; Gibco) supplemented with 10% FBS (10270106; Gibco), 1 g/l sodium bicarbonate (144-55-8; Sigma-Aldrich), 500 μg/ml G-418 solution (4727878001; Roche), and penicillin/streptomycin (15140122; Gibco) at 37°C and 5% CO_2_. Cells were used for a maximum of 20 passages.

### Cell seeding

50-mm^2^ glass coverslips were coated with 10 μg/ml of fibronectin (0895; Sigma-Aldrich) at 37°C for 30 min. Coverslips were then rinsed in PBS, and PDMS stencils were placed on top and gently pressed down to adhere. MDCK-E-cadherin-RFP cells were seeded at appropriate cell numbers for desired density within the PDMS wells and left to attach for 30–60 min before wells were flooded with DMEM. Cells were left to expand at 37°C and 5% CO_2_ overnight. The PDMS stencils were peeled off, and cells were either fixed according to the method described below 10 min after peeling (for the 0-h time point) or placed back in the incubator with fresh media for an additional 24 or 48 h before fixation (for the 24- and 48-h time points).

### Immunostaining

Cells were fixed with 4% formaldehyde (28906; Thermo Fisher Scientific) in PBS for 15 min at room temperature (RT). After fixation, cells were permeabilized with 0.2% Triton X (A16046; Alfa Aesar) in PBS for 5 min at RT. Cells were rinsed with PBS and then blocked with 3% BSA (A7906; Sigma-Aldrich) in PBS at RT for at least 45 min, or at 4°C overnight. For analysis of YAP, cells were incubated with primary mouse anti-YAP monoclonal Ab (1:100) (sc-101199; Santa Cruz) in 1% BSA in PBS overnight at 4°C. Cells were washed with 0.05% Tween (BP337-100; Thermo Fisher Scientific) in PBS 3 times for 1 min with gentle rocking and then incubated with 1:400 Alexa Fluor 488 goat anti-mouse secondary Ab; for cell count using nuclear signal, 1:500 Hoechst 33342 (62249; Thermo Fisher Scientific) and 1% BSA in PBS was added with the secondary Ab for 1 h at RT before mounting the coverslips on slides for imaging.

### Confocal microscopy

Images were acquired using a 20× (0.75 NA) (Plan Apo) objective on a Nikon Confocal A1RHD microscope, with 405-, 488-, and 561-nm laser lines and equipped with GaAsP PMT and PMT detectors. To capture the full cell volume, z-stacks with a step size of 1.5 µm were acquired. A custom Nikon JOBS script was used to acquire multipoint z-stacks across the whole colony.

### Bb washout

Cells were seeded on coverslips as previously described, and upon reaching the desired density, media were replaced with media containing 50 μM of Bb (203390; Merck) in PBS. After 60 min of Bb incubation, cells were rinsed in PBS and kept for a further 20 min in DMEM at 37°C before peeling the stencils and performing the experiments as previously described.

### Image analysis

The image analysis was carried out using Julia (version 1.10.4) (Bezanson et al, 2017). Each fluorescence channel, E-cadherin, nuclear, and YAP, was reduced to a 2D image via maximum intensity projection. The membrane channel was used as a reference for stitching partially overlapping fields-of-view. For segmentation, a global threshold (Rosin’s method) was first applied to identify the stencil foreground, and adaptive thresholding with multiple window sizes was then used on the nuclear channel to separate nuclei (Rosin, 2001). After thresholding, morphological filters and a watershed step were applied to improve the segmentation and separate adjacent nuclei. To segment the cytoplasmic region, the E-cadherin channel was used for thresholding, followed by a watershed algorithm to delineate each cell’s boundary around its nucleus (Bradley & Roth, 2007). Duplicate cells detected in overlapping regions were merged so that each cell was only counted once. To quantify YAP localization, nuclear YAP intensity was measured directly, whereas cytoplasmic YAP was calculated in a perinuclear annulus defined by a distance transform, selecting pixels within a set range from the nucleus. The mean intensity in each mask was used to compute a nuclear-to-cytoplasmic intensity ratio for each cell.

### Monolayer stress microscopy

PDMS substrates with elastic moduli of 3 and 15 kPa were prepared by mixing the components A and B of CY 52-276 (Dow Corning) at ratios of 6:5 and 1:1, respectively (Parker et al, 2021). The mixture was spread into 35-mm Petri dishes and cured at 80°C for 2 h. The cured gels were silanized with 10% (3-aminopropyl)triethoxysilane (Sigma-Aldrich) in absolute ethanol for 10–15 min, rinsed thoroughly with absolute ethanol three times, and air-dried. For traction force microscopy, substrates were coated with carboxylate-modified fluorescent microspheres (0.2 μm, F8810; Invitrogen) diluted 1:500 in Mq water. The bead solution was sonicated for 5 min before application. After a 5-min incubation, substrates were rinsed with Mq water three times, dried, and coated with 10 μg/ml human plasma fibronectin (in PBS) for 1–2 h at 37°C. Cells were seeded onto the functionalized substrates and cultured overnight to form a confluent monolayer before imaging.

Monolayers were imaged live on an inverted wide-field fluorescence microscope (Nikon Ti2-E) equipped with a 10× Plan Apo objective (NA 0.45), a CMOS camera (Nikon DS-Qi2), and an environmental chamber (Okolab) maintained at 37°C and 5% CO_2_. Phase-contrast images of cells and wide-field fluorescence images of embedded microspheres (filter cube: Ex. 543–566 nm, Em. 582–636 nm) were acquired simultaneously every 10 min. At the end of each time lapse, cells were detached by adding 200 μl of 10% Triton X-100 to the medium, and a reference fluorescence image of the relaxed bead layer was acquired. Fluorescent bead images were processed using FIJI for stabilization and illumination correction. Bead displacement fields were calculated by registering each time-lapse frame against the reference frame using particle image velocimetry in PIVlab (32 × 32-pixel interrogation windows with 50% overlap) (Thielicke & Stamhuis, 2014). Traction forces were computed from the displacement fields using Fourier transform traction cytometry, modelling the PDMS substrate as a linear elastic, isotropic half-space. Intercellular stress tensor fields within the monolayer were subsequently reconstructed using Bayesian inversion stress microscopy (Nier et al, 2016). The isotropic (hydrostatic) stress was calculated as (σxx + σyy)/2, where positive values denote tension and negative values compression. Analysis was performed on uncropped images with free boundary conditions. Afterwards, the boundary region of approximately 20% was excluded to avoid edge artefacts.

### Model

The simulations of cell colonies used an agent-based model (Li et al, 2021, 2022) with some modifications as described in Supplemental Data 1. Cells were modelled as soft discs sitting on a two-dimensional substrate. Each cell was bestowed with an idealized cell cycle, which is regulated by local pressure. The cell cycle in turn regulates the cell’s proliferation and growth dynamics. The cells’ positions evolve according to overdamped dynamics, because intercellular forces and substrate friction dominate inertial forces (for details, see Supplemental Data 1). The analysis of the simulation results was carried out in Python, making use of the NumPy and Matplotlib libraries.

Supplemental Data 1. Mechanical memory of confinement pressure governs expansion size in epithelial monolayers.

## Supplementary Material

Reviewer comments

## Data Availability

The data that support the findings in this study are available from the corresponding author upon reasonable request. The analysis pipeline code included is available at https://github.com/nordenfeltLab/stencil-module.
